# Perturb-Then-Diagonalize Vibrational Engine Exploiting Curvilinear Internal Coordinates

**DOI:** 10.1021/acs.jctc.2c00773

**Published:** 2022-11-02

**Authors:** Marco Mendolicchio, Julien Bloino, Vincenzo Barone

**Affiliations:** † Scuola Superiore Meridionale, Largo S. Marcellino 10, Napoli I-80138, Italy; ‡ Scuola Normale Superiore, Piazza dei Cavalieri 7, Pisa I-56126, Italy

## Abstract

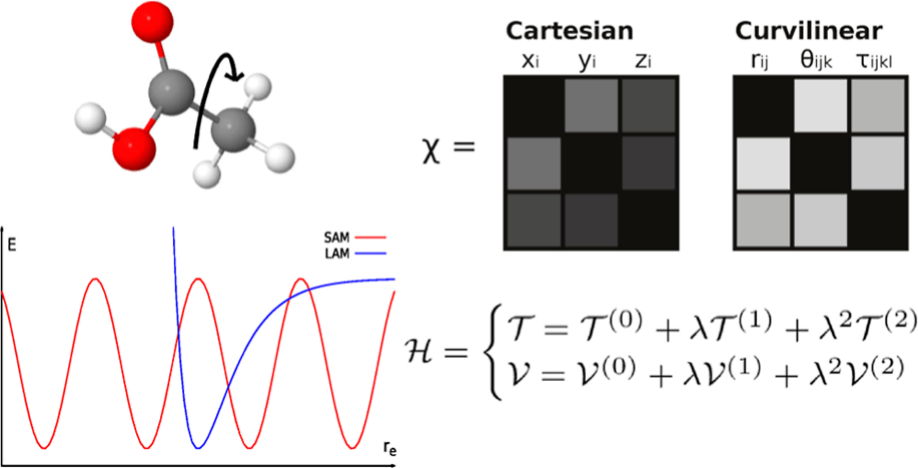

The present paper is devoted to the implementation and validation of a second-order perturbative approach to anharmonic vibrations, followed by variational treatment of strong couplings (GVPT2) based on curvilinear internal coordinates. The main difference with respect to the customary Cartesian-based formulation is that the kinetic energy operator is no longer diagonal, and has to be expanded as well, leading to additional terms which have to be taken into proper account. It is, however, possible to recast all the equations as well-defined generalizations of the corresponding Cartesian-based counterparts, thus achieving a remarkable simplification of the new implementation. Particular attention is paid to the treatment of Fermi resonances with significant number of test cases analyzed fully, validating the new implementation. The results obtained in this work confirm that curvilinear coordinates strongly reduce the strength of inter-mode couplings compared to their Cartesian counterparts. This increases the reliability of low-order perturbative treatments for semi-rigid molecules and paves the way toward the reliable representation of more flexible molecules where small- and large-amplitude motions can be safely decoupled and treated at different levels of theory.

## 1 Introduction

Thanks to significant developments in both software and hardware in the last few decades, computational spectroscopy has become an invaluable tool for both experimentally and theoretically oriented research works.^1,2^ In the specific case of vibrational and ro-vibrational spectroscopy, the basic rigid rotor/harmonic oscillator (RRHO) model is implemented in all major quantum chemistry programs. However, more sophisticate models are needed in several circumstances [e.g., high-resolution spectroscopy, large-amplitude motions (LAM), and so forth], which should possibly couple accuracy and feasibility for medium- to large-size systems.^3^

Among the different approaches available for going beyond the RRHO approximation,^4−28^ those based on low-order perturbation theory applied to the Watson Hamiltonian (i.e., a fourth-order polynomial expansion of the potential energy expressed in Cartesian normal modes) are particularly appealing for their remarkable cost/performance balance, at least for semi-rigid molecular systems.^29−40^

Moreover, a very general and robust model (referred to as GVPT2^32^) can be built, which involves the diagonalization of relatively small Hamiltonians coupling a reduced number of strongly interacting states and including the second-order perturbative contributions of all the other ones.^41,42^ A number of other models have been introduced (e.g., the so-called VPT2 + F^43^ and VPT2 + K^44^ methods), which can be seen as particular cases of the GVPT2 approach and allow, in principle, the inclusion of any type of coupling, irrespective of resonance conditions. Although the conventional implementations of these models employ different equations for spherical, linear, symmetric, and asymmetric tops,^45^ it has been recently shown that the canonical representation used for the development of VPT2 equations of asymmetric tops can be extended to linear and symmetric tops, provided that a series of a posteriori transformations are performed.^46^

Further improvements can be obtained resorting to higher-order (usually sextic) anharmonic force fields coupled with variational [e.g., vibrational configuration interaction (VCI)^6,8,14^] or more accurate (e.g., VPT4^47^) perturbative developments. Unfortunately, this kind of approaches converges slowly, and their cost becomes rapidly prohibitive as the dimension of the molecular system increases. An alternative route is based on reduced-dimensionality Hamiltonians tailored to describe a limited number of LAMs. Approaches belonging to this category are the internal coordinate path Hamiltonian (ICPH)^48^ and the reaction path Hamiltonian (RPH),^49−52^ aimed at describing single LAMs or the reaction surface Hamiltonian (RSH)^53^ and reaction volume Hamiltonian (RVH),^54^ for the case of two or three LAMs, respectively.

Whenever the couplings between small-amplitude motions (SAMs) and LAMs are small, the SAMs (and, possibly, the SAMs–LAMs couplings) can be treated by the GVPT2 model, whereas the sub-problem of LAMs can be solved, for instance, by the so-called discrete variable representation (DVR), which is a quasi-variational, numerical method, introduced by Light and co-workers^55^ and later re-derived by Colbert and Miller.^56^ Unfortunately, normal modes based on Cartesian coordinates often give rise to non-negligible couplings, whereas internal (curvilinear) coordinates can strongly reduce the couplings between different classes of vibrations.^57^ One major drawback of internal coordinates is that their definition is not unique, and their construction can be quite involved, especially when targeting medium-to-large systems. This problem is solved by the redundant set of internal coordinates composed of all the bond lengths, valence, and dihedral angles, which is uniquely defined by the molecular topology.^58,59^ Thus, the route is paved toward the development of a general and robust GVPT2 platform employing curvilinear coordinates.

The basic equations of VPT2 in curvilinear coordinates have been derived by Quade^60^ and reworked more recently by Isaacson.^57^ The main difference between rectilinear (Cartesian) and curvilinear (internal) coordinates stems from the expansion of the kinetic energy operator, which introduces additional, possibly resonant, terms. However, full re-derivation of the equations allowed us to recast them in terms of quite straightforward generalizations of those based on Cartesian coordinates, so that it has been possible to extend the already available GVPT2 engine to curvilinear coordinates. Of course, kinetic energy contributions must be computed, but this does not involve additional quantum chemical computations, so that GVPT2 remains extremely effective in this context as well. Since the new formulation incorporates the recent generalization of the asymmetric-top equations to non-Abelian groups,^46^ all kinds of molecules can be treated with the same formalism.

This paper is organized as follows. We start with a discussion of the main features of the new GVPT2 engine, emphasizing the differences and similarities with the well-known equations for Cartesian coordinates. A robust strategy for the identification and treatment of Fermi resonances is also presented, followed by some technical aspects of the general implementation. After sketching the essential computational details, a number of test cases are analyzed to validate the new engine for semi-rigid molecules and to define the most suitable routes for coupling accuracy with effectiveness. As expected, inter-mode couplings are significantly smaller for curvilinear internal coordinates than for their Cartesian counterparts, paving the way toward achieving effective models enforcing the separation between SAMs and LAMs. The main conclusions and most promising perspectives are given in the last section.

## 2 Theory

### 2.1 Framework

The simplest set of internal coordinates is represented by the so-called primitive internal coordinates (PICs), which are composed of all bond lengths, valences, and dihedral angles and are uniquely defined by the molecular topology.^61,62^ While this set is generally redundant, this does not cause any problem (in analogy with translation and rotations when employing Cartesian coordinates) since all eigenvectors with vanishing eigenvalues can be removed after the harmonic problem is solved (vide infra). Next, PICs can be expressed in terms of their Cartesian counterparts by means of a Taylor series expansion
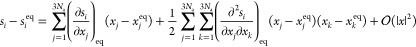
1where *N*_a_ is the number of atoms, ***s*** is the vector containing the internal coordinates, whose values at the equilibrium geometry are collected in the vector ***s***^eq^, and ***x*** contains the atomic Cartesian coordinates. When the interest is focused on relatively low-vibrational excitations (i.e., close to the bottom of the potential energy surface (PES) well), eq 1 can be safely truncated at the second order and rewritten in a more compact form
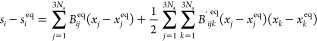
2

The elements of the so-called Wilson **B** matrix^63^ and its first derivative, **B**′, are
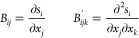
3and have well-known analytical expressions.^61^ By construction, only the indices *j* and *k* of the **B**′ tensor commute, whereas the **B** matrix is not symmetric or necessarily square, since the number of internal coordinates can be different from that of Cartesian coordinates.

### 2.2 Vibrational Hamiltonian in Curvilinear Coordinates

The starting point of the derivation is the definition of the expression of the kinetic-energy operator *T* in terms of the so-called Wilson **G** matrix
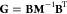
4where **M** is the diagonal matrix of the nuclear masses, while **B** is defined in eq 3. As a result, the vibrational kinetic energy  is given by^64−66^
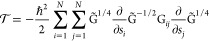
5where . A more convenient form of eq 5 has been proposed by Podolosky,^64^ further discussed by Lauvergnat,^66^ and re-derived in this work (see Section S1 of the Supporting Information), leading to the following expression
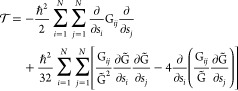
6where the last term corresponds to a purely quantum-mechanical contribution to the kinetic energy, usually referred to as the extra-potential term.^67^Equation 6 represents the kinetic energy operator in terms of curvilinear coordinates ***s***. However, application of perturbation theory to solve the vibrational problem requires a set of suitable reference wave functions too. In analogy with the treatment based on Cartesian coordinates, the harmonic oscillator model is employed to this end, by means of the so-called Wilson GF method (see Section S2 of the Supporting Information),^68^
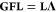
7where **F** is the Hessian matrix of the potential energy with respect to the internal coordinates, **L** is the matrix containing the normal coordinates, and **Λ** is the diagonal matrix of squared harmonic frequencies (ω).

One of the advantages of a polynomial expansion in the normal-mode basis is that it leads to analytic integrals for both coordinate and momentum operators, together with a particularly simple second-quantization formulation, with these features strongly simplifying the identification of non-vanishing contributions in the perturbative expansion.

Equation 6 can be rewritten in terms of the dimensionless normal coordinates ***q*** and their conjugate momenta ***p***
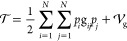
8where **g**, , and  are the **G** matrix, its determinant, and the extra-potential term expressed in wavenumbers, respectively (see Section S3 of the Supporting Information)
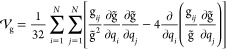
9

The potential energy (expressed in terms of dimensionless coordinates ***q***) must be added to the kinetic energy in order to complete the vibrational Hamiltonian . Since the extra-potential term is well approximated by its value at the equilibrium configuration,^57,69^ it does not play any role in the calculation of transition energies. As a consequence, it will be neglected from now on, leading to the following expression of the vibrational Hamiltonian
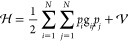
10

### 2.3 Perturbative Expansion of the Vibrational Hamiltonian

The perturbative treatment of  is carried out by expanding both the kinetic and potential energies up to the second order. From here on, the symbol  will be used to indicate the first term of eq 10, so that
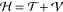
11

The **g** matrix entering the kinetic energy expression can be expanded to the second order
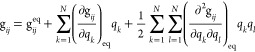
12where g_*ij*_^eq^ = ω_*i*_δ_*ij*_ at the equilibrium configuration (see Section S3 of the Supporting Information) and δ_*ij*_ is the Kronecker delta.

By inserting eq 12 in the definition of  and introducing the following notation
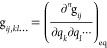
13the kinetic energy can be written as a perturbative series
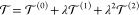
14where
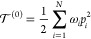
15
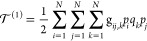
16
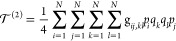
17

We recall that only the first and second indices of  commute, while *i* only commutes with *j* and *k* only commutes with *l* in .

The expansion of the potential energy is analogous to its Cartesian counterpart
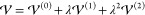
18where  is the harmonic potential, while  and  contain, respectively, the cubic- and quartic-order contributions to the PES
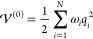
19
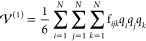
20
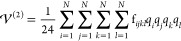
21with
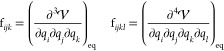
22

The only difference with respect to Cartesian coordinates is the absence of the Coriolis term and the form of normal modes, which are now expressed in terms of internal (curvilinear) coordinates.

The full vibrational Hamiltonian can be written as follows
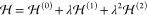
23where
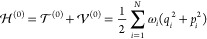
24
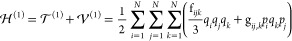
25
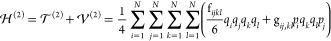
26

The curvilinear coordinate version of VPT2 requires not only the cubic and quartic force constants but also the first and second derivatives of the **g** (or **G**) matrix, whose calculation will be discussed in Section 3.

### 2.4 Vibrational Energies

In analogy with the treatment based on Cartesian coordinates, the anharmonic energies can be obtained through either canonical van Vleck (CV) or Rayleigh–Schrödinger (RS) perturbation theory (PT), which lead to the same final expressions. As already mentioned, the main difference with respect to the Cartesian-based framework is the presence, together with potential energy contributions, of additional terms arising from the kinetic energy. In order to clarify this point, let us recall the expression of the energy of the *R*th vibrational state expanded up to the second order
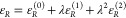
27

The form of the harmonic Hamiltonian  is equivalent in Cartesian- and curvilinear-based formulations, so that both eigenvalues and eigenvectors are given by the customary expressions, and the first-order correction to the energy (eq 25) always vanishes
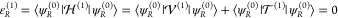
28since both  and  are odd operators in terms of normal coordinates and their conjugate momenta.

Finally, the second-order correction to the energy, ε_*R*_^(2)^, is given by
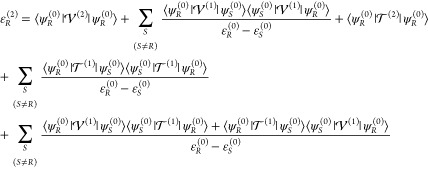
29

Inspection of eq 29 shows that the anharmonic correction to each energy level is composed of three contributions, namely, potential (first line), kinetic (second line), and a cross term (third line). In the Cartesian version, only the potential term (albeit including Coriolis contributions) is present, so that the development becomes more complex when employing curvilinear internal coordinates. In order to accelerate the development stage, as well as reduce the possibility of errors, the derivation of ε_*R*_^(2)^ has been carried out by a multi-step procedure, with the help of ad hoc programs employing the second-quantization formalism followed by a manual post-processing.

The final expression of ε_*R*_ can be recast in the customary form
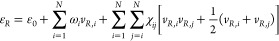
30where ***v***_*R*_ represents the vector of vibrational quantum numbers for the *R*-th state and ε_0_ is the zero-point vibrational energy (ZPVE), which will not be considered in the following because it does not affect energy differences between vibrational states. The **χ** matrix is given by the sum of three distinct contributions
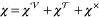
31where the superscripts , , and × indicate the potential, kinetic, and a cross term, respectively. The form of the potential contribution is the same for Cartesian and curvilinear coordinates (see Section S4 of the Supporting Information), except for the presence of Coriolis contributions in the former case
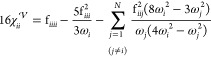
32
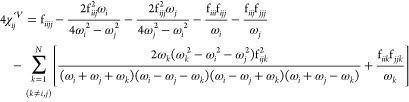
33while the purely kinetic contribution is
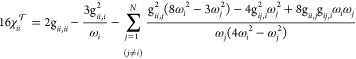
34
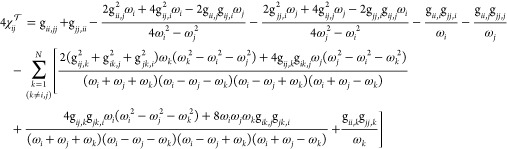
35

Finally, the cross term is
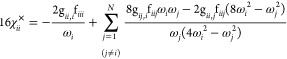
36
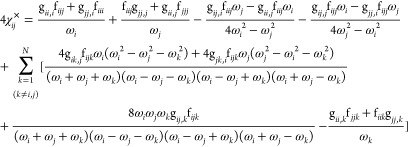
37

While the above expressions (fully equivalent to those reported in refs (57) and (60)) permit us to obtain the transition energies, a further algebraic manipulation leads to a more convenient form. By applying the partial fraction decomposition to eq 32 through eq 37 (see Section S5 of the Supporting Information for more details) and introducing the tensors η_*ijkl*_, σ_*ijk*_, and ρ_*ijk*_, we get
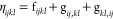
38
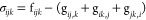
39
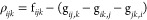
40

Equation 31 can be rewritten as
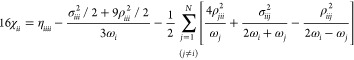
41
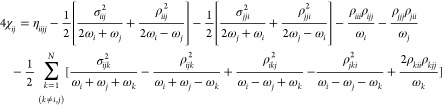
42

Comparison of eqs 41 and 42 with their Cartesian counterparts (see eqs S33 and S34 of the Supporting Information) shows that the general form of the **χ** matrix does not change. More specifically, by reintroducing the Coriolis contribution and setting the derivatives of the **g** matrix to zero (η_*ijkl*_ = f_*ijkl*_ and σ_*ijk*_ = ρ_*ijk*_ = f_*ijk*_) in eqs 41 and 42, S36 and S37 are recovered. A similar procedure can be carried out to perform the inverse transformation. Note that, while the Coriolis term is absent in the internal-based VPT2 Hamiltonian, the perturbative development of the kinetic energy operator yields contributions formally equivalent to it. Therefore, the internal-based expression can be interpreted as a generalization of the Cartesian-based one. This formal equivalence presents two main advantages:

- Implementation of eqs 41 and 42 into an existing code based on the Cartesian-based formulation is quite straightforward;
- Analysis of Fermi resonances, which is the object of the next section, can be directly extended to curvilinear coordinates.

### 2.5 Fermi Resonances

Equations 41 and 42 show that the calculation of energy levels at the VPT2 level is plagued by Fermi resonances, irrespective of the use of rectilinear or curvilinear coordinates.^38^ Furthermore, the form of the perturbed vibrational Hamiltonian  (eq 23) does not affect the nature of the contact transformation. As a consequence, the definition of the interaction terms of the contact-transformed Hamiltonian between interacting states can be directly generalized to the use of curvilinear coordinates. This premise is of fundamental importance for the analysis of Fermi resonances, the redefinition of suitable tests for their detection, and the variational correction at the basis of the GVPT2 approach.

#### 2.5.1 Internal-Based Contact-Transformed Vibrational Hamiltonian

The off-diagonal elements of the contact-transformed Hamiltonian  between two interacting states  and  can be written in terms of different orders of the original Hamiltonian .^44^
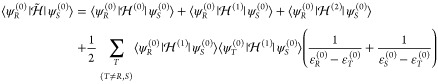
43

In analogy with the expressions for the energy levels, the interaction element (eq 43) can also be partitioned into three contributions, which arise from the insertion of eq 23 in the above expression
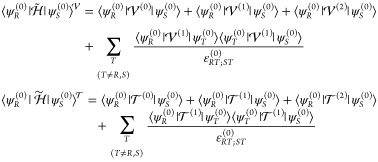
44
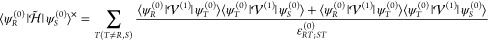
45where the term 
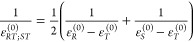
46has been introduced for the sake of readability. Let us recall that one of the advantages of separating the contributions of different terms relies on the fact that the potential term is formally equivalent, except for the Coriolis couplings, to the expression derived in the Cartesian-based formulation. Equation 44 has been used to derive the interaction terms corresponding to Fermi resonances, which are discussed in the next section.

#### 2.5.2 Diagnostic of Fermi Resonances: Extension of the Martin Test

The close correspondence between the **χ** matrix for different sets of coordinates allows a straightforward extension to curvilinear coordinates of the so-called Martin test for the identification of Fermi resonances.^70^ By switching back to Dirac’s notation, the matrix elements coupling the states |***v***_*R*_ + 1_*k*_⟩ with |***v***_*R*_ + 2_*i*_⟩ or |***v***_*R*_ + 1_*i*_ + 1_*j*_⟩ are
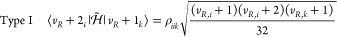
47
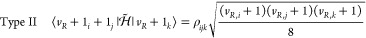
48which are obtained from the corresponding Cartesian-based expressions replacing f_*iik*_ and f_*ijk*_ by ρ_*iik*_ and ρ_*ijk*_, respectively. As a consequence, for all kinds of coordinates, the identification of Fermi resonances can be carried out by the same two-step procedure relying on the thresholds Δω^1–2^ and *K*^1–2^ with default values of 200 and 1 cm ^–1^, respectively.^71^

Once the set of Fermi resonances has been identified, the corresponding terms in eqs 41 and 42 are discarded, and the resulting **χ** matrix is used for the calculation of the energy levels within the so-called deperturbed (DVPT2) scheme. The interaction terms corresponding to Fermi resonances can be treated in a successive variational step (leading to a model broadly referred to as GVPT2) by diagonalizing small matrices, whose diagonal elements are the deperturbed energies, while off-diagonal elements can be obtained from eqs 47 and 48 (Table 1).

**Table 1:** Formulation of the Test for the Identification of Fermi Resonances in Both Cartesian- and Internal-Based Formulations of VPT2

	type I	type II
step 1^a^	|2ω_*i*_ – ω_*k*_| ≤ Δω^1–2^	|ω_*i*_ + ω_*j*_ – ω_*k*_| ≤ Δω^1–2^
step 2^b^	ρ_*iik*_^4^/256|2ω_*i*_ – ω_*k*_|^3^ ≥ *K*^1–2^	ρ_*iik*_^4^/64|ω_*i*_ + ω_*j*_ – ω_*k*_|^3^ ≥ *K*^1–2^

a Step 1 is the same regardless of the formulation of VPT2.

b ρ_*ijk*_ = *f*_*ijk*_ in the Cartesian-based VPT2 framework.

## 3 Implementation

The implementation of the new engine can be split into three main steps. In the first one, the set of internal coordinates is defined starting from the reference geometry and used to build the **B** matrix and the **B**′ tensor, with the former being also used to calculate the **G** matrix. To this end, we have implemented a new code for the analytical computation of **B**, **B**′, **G**, and **G**′ matrices for bond lengths, valences (linear and non-linear), and dihedral angles. It is also possible to use different curvilinear coordinates by reading the **B** and **B**′ matrices generated by other programs. In both cases, the first derivative g_*ij*,*k*_ can be simply computed from G_*ij*,*k*_ (see Section S3 of the Supporting Information), with the latter being given by
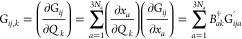
49where **G**′ is the tensor collecting the first Cartesian derivatives of the Wilson **G** matrix and can be further expanded by introducing the **B**′ tensor,
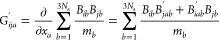
50which, in matrix form, becomes
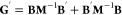
51

The terms g_*ij*,*kl*_ are obtained from their mass-weighted counterparts G_*ij*,*kl*_ using an expression similar to eq 49
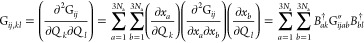
52

In the above expression, **G**^″^ collects the second-order Cartesian derivatives of the **G** matrix,
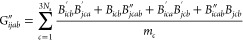
53in matrix form, it becomes
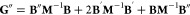
54where **B**^″^ is the second-order Cartesian derivative of the **B** matrix. Consequently, the analytical calculation of the terms G_*ij*,*kl*_ relies on the four-dimensional tensor **B**^″^, which presents some difficulties. In the first place, it is composed of all third-order derivatives of the internal coordinates with respect to Cartesian coordinates, whose derivation and implementation involve a significant effort. Furthermore, the use of the four-dimensional tensor **B**^″^ with one dimension equal to *N* and the other three equal to 3*N*_a_ may imply additional concerns in terms of both computer time and memory storage. For these reasons, a more viable strategy is the analytical computation of the first derivatives G_*ij*,*k*_, followed by their use in the finite-difference calculation of second derivatives.

The second step is the definition of the displacements along the curvilinear normal modes, the computation of Hessians at these geometries, and the assembly of potential and kinetic contributions to cubic and quartic force constants. This task is performed by a script, which calls an external quantum chemical package to compute the gradients and Hessians in Cartesian coordinates at suitable geometries. An external implementation has the advantage that the most computer-intensive (but embarassingly parallel) step can be performed in the most effective way, namely, distributed among different computing nodes. The calculation of the Hessian matrix **F** in internal coordinates can be carried out using the following expression^72^
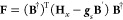
55where the internal-based gradient ***g***_*s*_ can be easily obtained starting from its Cartesian counterpart ***g***_*x*_ as
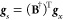
56

Furthermore, the overall contribution due to translations and rotations can be factored out by replacing (**H**_*x*_ – ***g***_*s*_**B**′) and ***g***_*x*_ by **P**(**H**_*x*_ – ***g***_*s*_**B**′)**P** and **P*g***_*x*_, respectively, where **P** = **B**^†^**B** represents the projection matrix.

Second derivatives of the **G** matrix are also obtained from finite-difference expressions
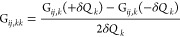
57
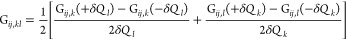
58

Equation 57 includes, of course, the terms G_*ii*,*kk*_ and G_*ii*,*ii*_, while eq 58 includes G_*ij*,*ij*_, G_*ii*,*kl*_ and G_*ij*,*kj*_.

As mentioned above, these computations have been always performed by a new script preparing the input stream and submitting harmonic computations for the different geometries needed in the finite-difference evaluation. Although different electronic structure codes could be employed in this step, all the computations reported in this work have been performed by the G16 package.^73^ Atomic units are used systematically together with angles in radians. On the basis of previous experience and several new numerical tests, a default step (δ*Q*) of 0.02 amu^1/2^ Bohr has been chosen for all kinds of coordinates.

The third step involves the implementation of the GVPT2 equations for curvilinear coordinates discussed in Section 2. This has been accomplished by extending the general platform for Cartesian coordinates already available in the Gaussian code.

**Figure 1:**
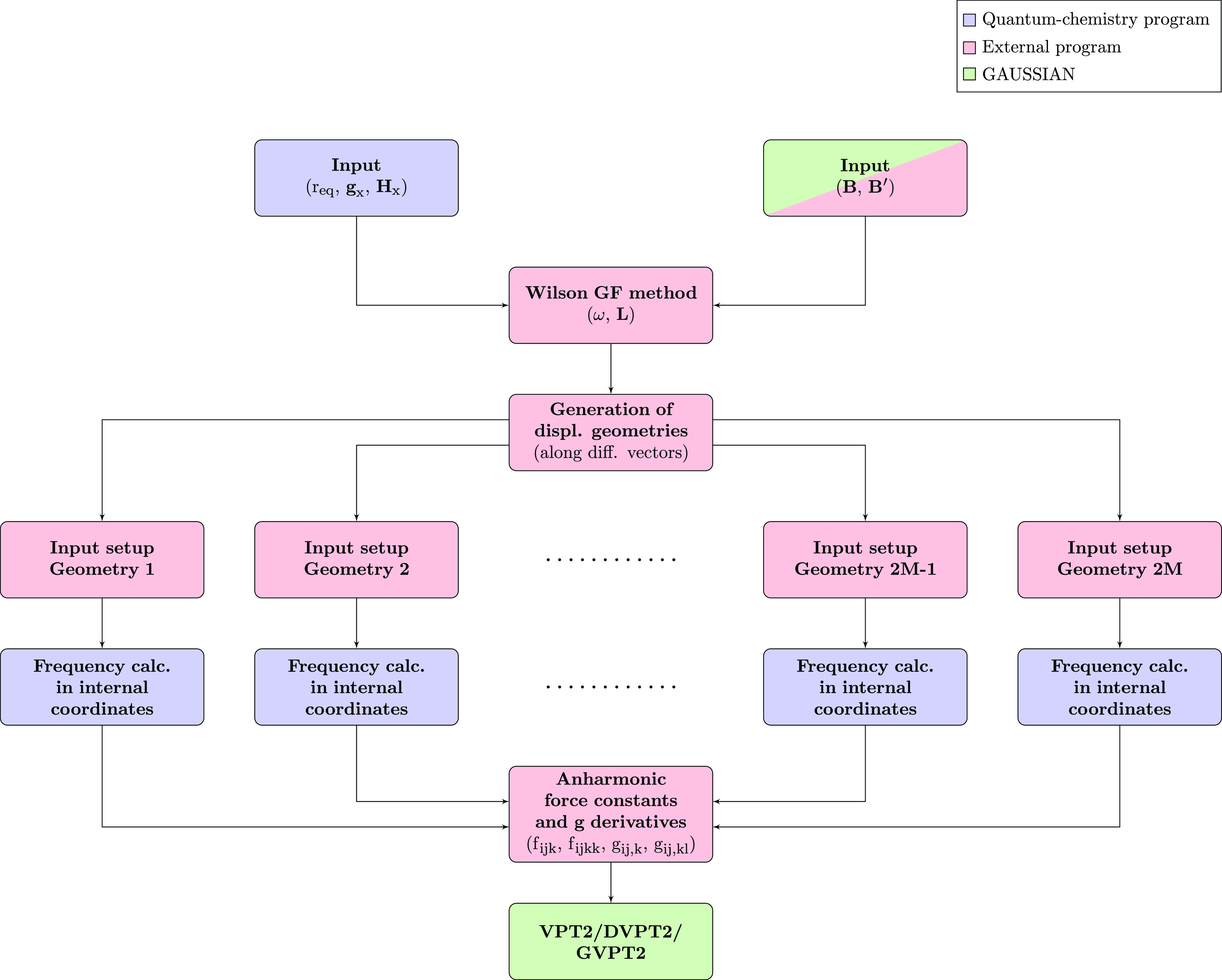
Flowchart describing the new workflow for the anharmonic calculations in curvilinear coordinates, where the tasks performed by a generic quantum chemical code, the Gaussian package, and the novel external program are highlighted. *M* represents the number of active modes.

A flowchart describing the whole workflow is sketched in Figure 1.

## 4 Computational Details

In light of previous experience, hybrid density functionals B3PW91^74^ and PW6B95^75^ were used in conjunction with the jul-cc-pVDZ (hereafter julDZ) basis set,^76^ whereas double-hybrid functionals B2PLYP^77,78^ and revDSD-PBEP86^79^ together with second-order Møller–Plesset PT (MP2)^80^ were employed in conjunction with the jun-cc-pVTZ (hereafter junTZ) basis set.^76^ Furthermore, empirical dispersion contributions were systematically added in DFT computations by means of Grimme’s D3 model with Becke–Johnson damping.^81,82^ The above computational levels will be denoted in the following as B3, PW6, B2, rDSD, and MP2, respectively.

## 5 Results and Discussion

**Figure 2:**
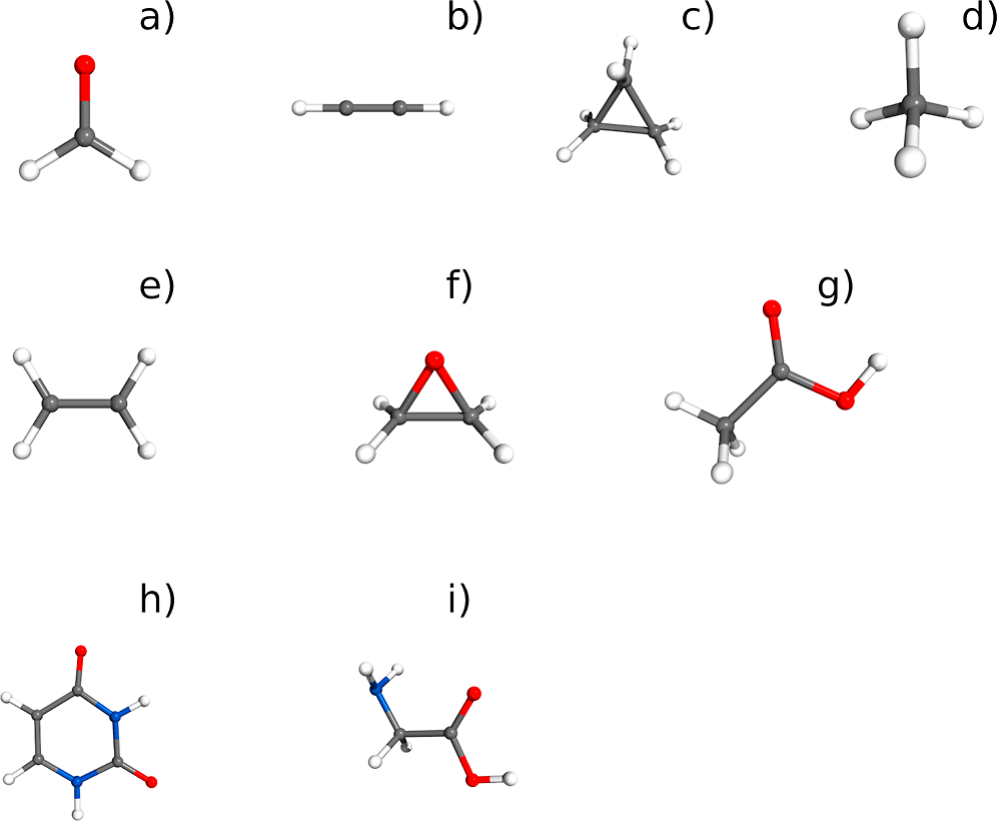
Structures of all the studied molecules. (a) Formaldehyde, (b) acetylene, (c) cyclopropane, (d) methane, (e) ethylene, (f) oxirane, (g) acetic acid, (h) uracil, and (i) Ip conformer of glycine.

In this section, we will present a number of results obtained by the new VPT2 engine with the objective of validating its implementation and highlighting the advantages of curvilinear over Cartesian coordinates concerning both effectiveness and accuracy. After considering semi-rigid systems, where different sets of coordinates provide comparable results (but the number and strength of inter-mode couplings are very different), we will consider some prototypical flexible systems, where the advantages of curvilinear coordinates become more apparent. The structures of all the studied molecules are sketched in Figure 2.

### 5.1 Validation of VPT2 in Curvilinear Coordinates

The new VPT2 implementation has been validated for the asymmetric (formaldehyde), linear (acetylene), symmetric (cyclopropane), and spherical (methane) tops shown in Figure 2a–d. Comparison between VPT2 results in Cartesian and curvilinear coordinates permits us to test the correctness of both the VPT2 equations (also in the presence of Fermi resonances) and the elements of the **G** matrix and its derivatives. All the computations have been performed at the MP2/junTZ level, which couples semi-quantitative accuracy with the lack of any numerical noise, as it would be the case for DFT methods. Note that, in the absence of numerical errors, harmonic frequencies are identical for any set of coordinates.

The results collected in Tables 2–5 show that for small semi-rigid molecules Cartesian and curvilinear coordinates provide equivalent results, irrespective of the symmetry (Abelian or non-Abelian point group) of the system. Furthermore, in the case of formaldehyde, Coriolis couplings are not negligible, especially for the wagging and CH_2_ asymmetric stretching, and the curvilinear results are much closer to the Cartesian counterparts including Coriolis couplings than to those neglecting them (see Table 2). This shows that some terms in the development of kinetic energy in curvilinear coordinates are equivalent to Coriolis couplings in Cartesian coordinates.

**Table 2:** Comparison of the Cartesian and Curvilinear VPT2, DVPT2, and GVPT2 Wavenumbers (in cm^–1^) of Formaldehyde at the MP2/junTZ Level

				Cartesian			curvilinear		
	assignment	symm.	ω	*ν*_VPT2_^a^	*ν*_DVPT2_	*ν*_GVPT2_	*ν*_VPT2_	*ν*_DVPT2_	*ν*_GVPT2_
1	CH_2_ s str.	A_1_	2975	2829 (2827)	2820	2829	2829	2829	2829
2	C=O str		1756	1724 (1723)	1724	1724	1724	1724	1724
3	HCH s bend		1545	1512 (1510)	1512	1512	1512	1512	1512
4	HCH op wag	B_1_	1203	1183 (1169)	1183	1183	1183	1183	1183
5	CH_2_ a str.	B_2_	3051	3029 (3017)	2897	2866	3029	2902	2869
6	HCH a bend		1271	1250 (1246)	1250	1250	1250	1250	1250
2 + 6	comb. band		2975	2835	2967	2999	2835	2962	2995

a In parenthesis, the VPT2 frequencies obtained without including Coriolis couplings have been reported.

**Table 3:** Comparison of Cartesian and Curvilinear VPT2 Fundamental Wavenumbers (in cm^–1^) of Acetylene at the MP2/junTZ Level

			Cartesian	curvilinear
assignment	symmetry	ω	*ν*_VPT2_	*ν*_VPT2_
CH s str.	Σ_*g*_	3525	3397	3397
CC str		1969	1931	1931
CH a str	Σ_*u*_	3437	3317	3317
HCC s bend	Π_*g*_	592	609	609
HCC a bend	Π_*u*_	748	739	739

**Table 4:** Comparison of the Cartesian and Curvilinear VPT2, DVPT2, and GVPT2 Fundamental Wavenumbers (in cm^–1^) of Cyclopropane at the MP2/junTZ Level

			Cartesian			curvilinear		
assignment	symmetry	ω	*ν*_VPT2_	*ν*_DVPT2_	*ν*_GVPT2_	*ν*_VPT2_	*ν*_DVPT2_	*ν*_GVPT2_
CH_2_ s str.	A_1_^′^	3196	3075	3075	3075	3074	3074	3074
CH_2_ sciss.		1533	1504	1485	1515	1501	1484	1506
ring str.		1231	1203	1203	1203	1201	1201	1201
CH_2_ twist	A_1_^″^	1166	1131	1131	1131	1128	1128	1128
CH_2_ wagg.	A_2_^′^	1085	1057	1057	1057	1054	1054	1054
CH_2_ a str.	A_2_^″^	3298	3154	3154	3154	3154	3154	3154
CH_2_ rock.		869	863	863	863	857	857	857
CH_2_ s str.	E′	3187	3067	3067	3068	3067	3067	3067
CH_2_ def.		1485	1440	1444	1443	1436	1439	1440
CH_2_ wagg.		1050	1022	1022	1022	1017	1017	1017
ring def.		905	878	878	878	876	876	876
CH_2_ a str.	E^″^	3279	3135	3135	3135	3135	3135	3135
CH_2_ twist + rock		1220	1192	1192	1192	1190	1190	1190
twist + rock.		747	741	741	741	734	734	734

**Table 5:** Comparison of the Cartesian and Curvilinear VPT2 Fundamental Wavenumbers (in cm^–1^) of Methane at the MP2/junTZ Level

			Cartesian	curvilinear
assignment	symmetry	ω	*ν*_VPT2_	*ν*_VPT2_
CH str.	A_1_	3073	2953	2953
bend.	E	1586	1549	1549
CH str.	T_2_	3209	3074	3074
bend		1352	1318	1318

### 5.2 Reconciling Accuracy and Feasibility

For small semi-rigid molecules, the accuracy of state-of-the-art quantum-chemical methodologies can rival that of experimental techniques.^83−85^ However, their extension to large (possibly flexible) systems faces a number of difficulties ranging from the very unfavorable scaling of such methods with the number of basis functions to the proper description of flat PESs.^3,86^ A viable route to obtain accurate results, even for relatively large molecular systems (a few dozens of atoms), is provided by dual-level models, which combine accurate calculations of molecular structures and harmonic force fields to cheaper yet reliable approaches for taking into account anharmonic contributions resulting from SAMs and, possibly, a small number of LAMs. The role of curvilinear coordinates in improving these aspects is analyzed in the next subsections.

### 5.3 Coupling Issue

**Figure 3:**
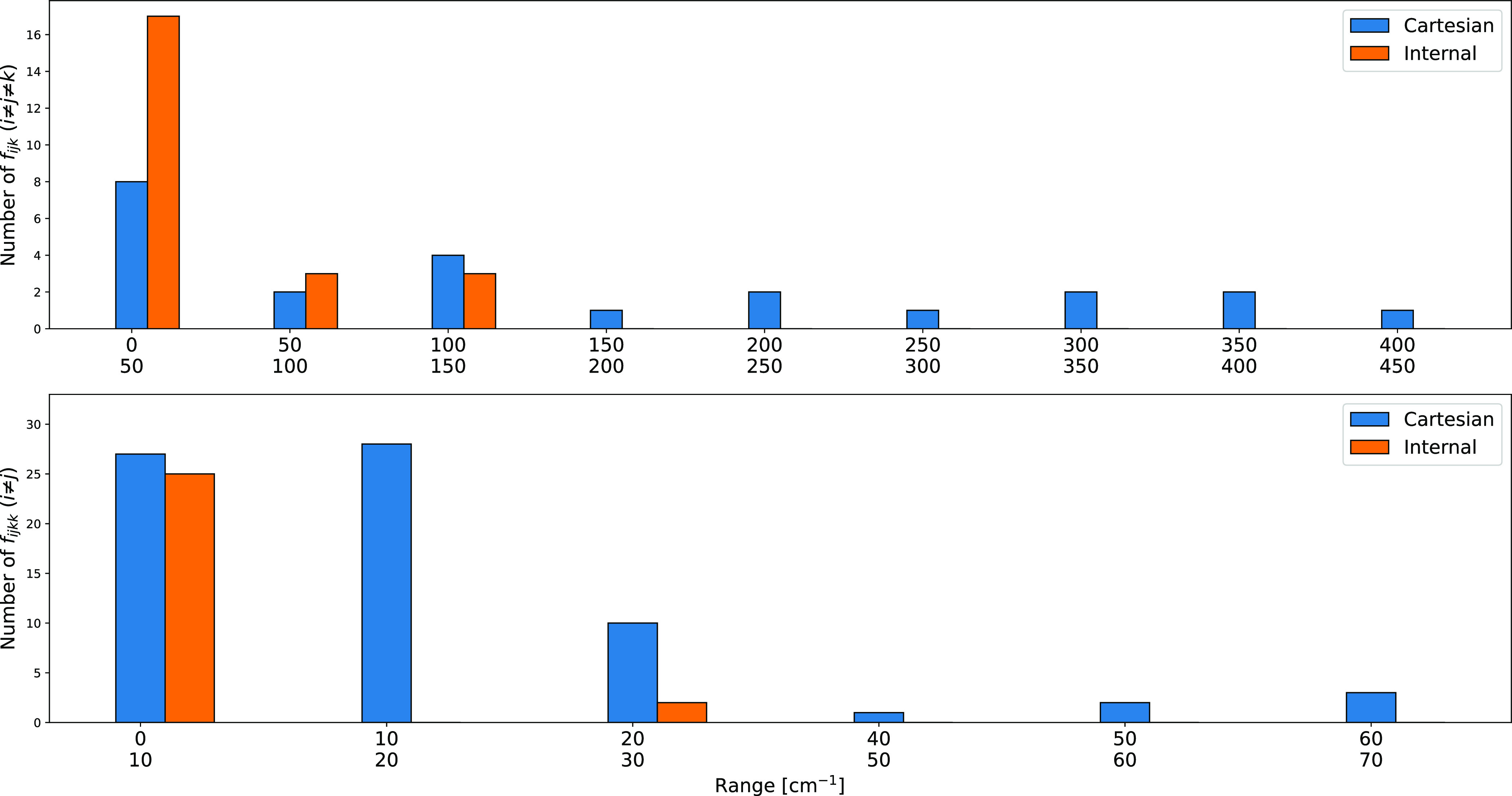
Comparison of the number of cubic (f_*ijk*_ (*i* ≠ *j* ≠ *k*)) and quartic (f_*ijkk*_ (*i* ≠ *j*)) force constants of ethylene above a given threshold (in cm^–1^) computed at the MP2/junTZ level with Cartesian or curvilinear coordinates.

The accuracy of low-level perturbative treatments is, of course, related to the number and strength of couplings between different modes and, especially, to the relative role played by two- and three-mode interactions. We will use ethylene (see Figure 2e) to analyze this aspect. As a matter of fact, GVPT2 results obtained employing Cartesian or curvilinear coordinates are virtually indistinguishable (as expected for semi-rigid molecules), but the number and strength of couplings determining the final result are different in the two cases. Furthermore, the terms neglected in VPT2 energies but actually computed in the numerical differentiation of analytical Hessians (i.e., three-mode quartic force constants) are significantly different in the two implementations. This is well evidenced in Figure 3, which shows that both the number and strength of all three-mode couplings are strongly reduced when using curvilinear internal coordinates.

**Figure 4:**
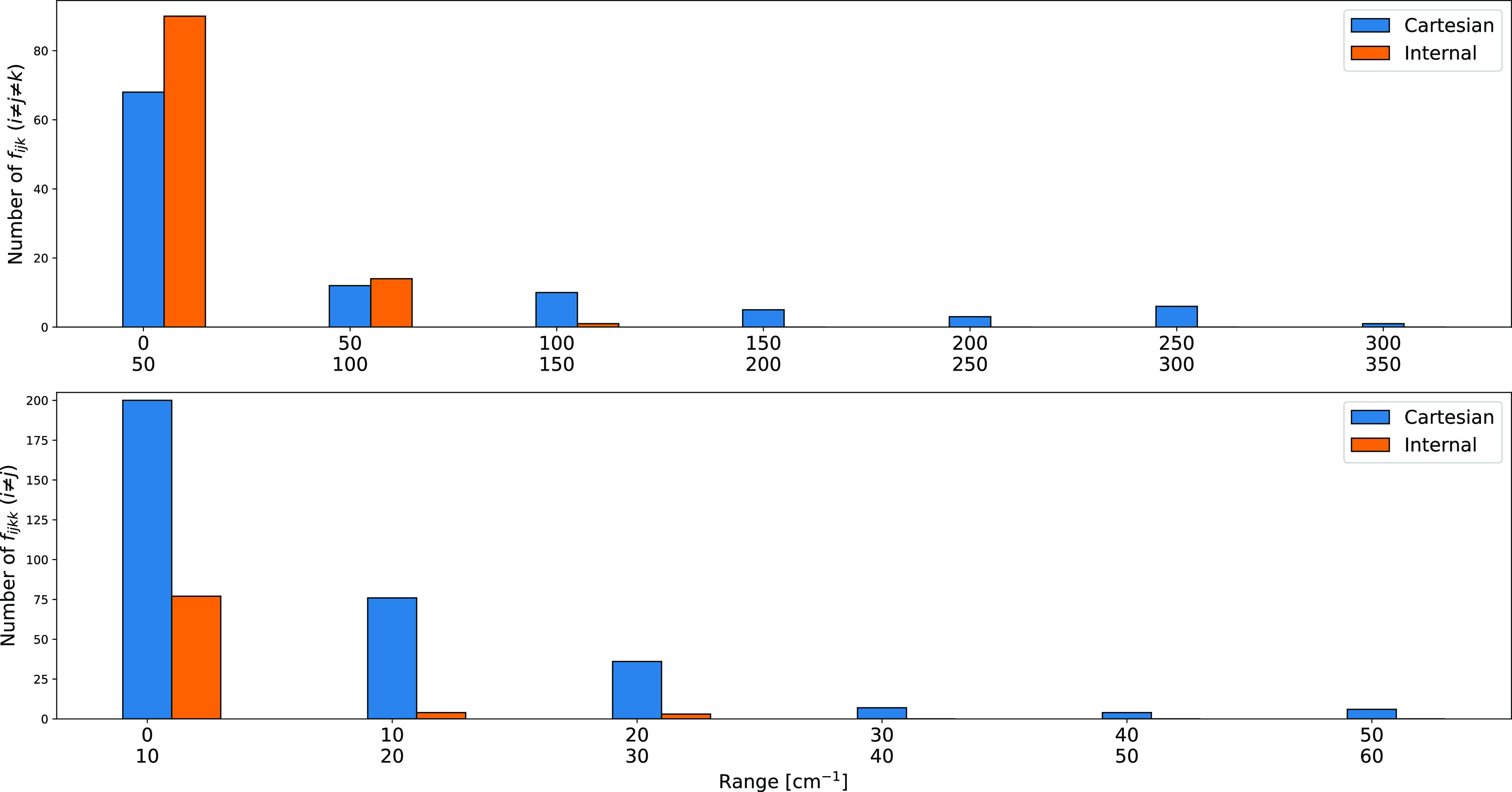
Comparison of the number of cubic (f_*ijk*_ (*i* ≠ *j* ≠ *k*)) and quartic (f_*ijkk*_ (*i* ≠ *j*)) force constants of oxirane above a given threshold (in cm^–1^) computed at the MP2/junTZ level with Cartesian or curvilinear coordinates.

Another example is offered by oxirane (see Figure 2f), whose computed vibrational energies are collected in Table 6. While more accurate results can be obtained increasing the computational level, all the experimental trends are correctly reproduced and, once again, the use of curvilinear coordinates strongly reduces the role of three-mode couplings (see Figure 4), which can thus be safely neglected with a few exceptions.

**Table 6:** Comparison of the Cartesian and Curvilinear VPT2, DVPT2, and GVPT2 Wavenumbers (in cm^–1^) of Oxirane at the MP2/junTZ Level with the Experimental Data

				Cartesian			curvilinear			
	assign.	symm.	ω	*ν*_VPT2_	*ν*_DVPT2_	*ν*_GVPT2_	*ν*_VPT2_	*ν*_DVPT2_	*ν*_GVPT2_	exp.^a^
1	(CH_2_ s-str)	A_1_	3160	3057	3030	3058	3057	3034	3057	3006
2	(CH_2_ scis)		1552	1503	1503	1503	1504	1504	1504	1498
3	(ring str)		1310	1279	1279	1279	1279	1279	1279	1271
4	(CH_2_ wag)		1155	1125	1125	1125	1125	1125	1125	1120
5	(ring def.)		902	880	880	880	880	880	880	877
6	(CH_2_ a-str)	A_2_	3264	3119	3119	3119	3119	3119	3119	3065
7	(CH_2_ twist)		1175	1151	1151	1151	1151	1151	1151	1142
8	(CH_2_ rock)		828	815	815	815	816	816	816	822
9	(CH_2_ s-str)	B_1_	3153	3045	3014	3050	3045	3021	3048	3006
10	(CH_2_ scis)		1519	1480	1480	1480	1480	1480	1480	1472
11	(CH_2_ wag)		1171	1143	1143	1143	1143	1143	1143	1151
12	(ring def)		851	822	822	822	822	822	822	892
13	(CH_2_ a-str)	B_2_	3250	3106	3106	3106	3106	3106	3106	3063
14	(CH_2_ twist)		1186	1163	1163	1163	1164	1164	1164	1142
15	(CH_2_ rock)		1059	1032	1032	1032	1033	1033	1033	822
2 + 2	overtone	A_1_	3103	2979	3005	2977	2980	3003	2979	
2 + 10	comb. band		3070	2951	2981	2945	2952	2976	2949	

a Ref (87).

**Figure 5:**
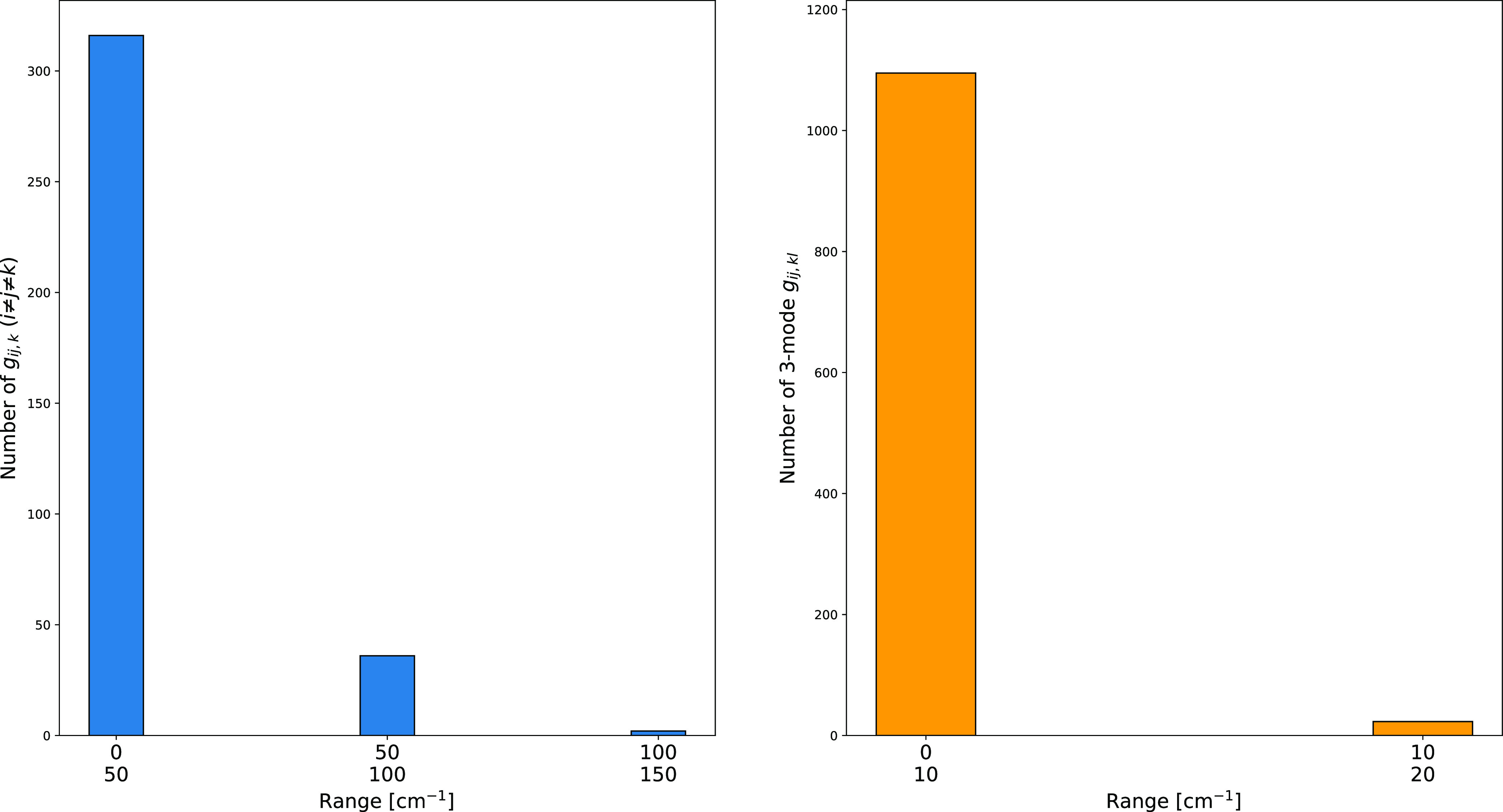
Number of three-mode first- and second-order **g** matrix derivatives of oxirane above a given threshold (in cm^–1^) computed at the MP2/junTZ level with curvilinear coordinates.

While only potential couplings involve an increased computational cost of the underlying electronic computations, a fully unbiased comparison between Cartesian and curvilinear implementations requires the evaluation of the role of kinetic couplings. Figure 5 shows that, as expected, three-mode kinetic contributions are essentially negligible.

An even more vexing problem is related to the presence of LAMs like, for example, methyl rotations. The situation is illustrated in Table 7 for the specific example of acetic acid (see Figure 2g). Although VPT2 results are very close for different sets of coordinates, the Cartesian description shows comparable contributions from the one-dimensional anharmonicity of the CH_3_ rotation and its coupling with other modes. As a consequence, any separation between LAMs and SAMs faces against severe difficulties. For example, neglecting inter-mode couplings, the computed frequency of CH_3_ torsion becomes completely unrealistic when employing Cartesian coordinates (−5213 cm^–1^), whereas the value issuing from curvilinear coordinates (68 cm^–1^) remains reasonable.

**Table 7:** Comparison of the Cartesian and Curvilinear GVPT2 Fundamental Wavenumbers (in cm^–1^) of Acetic Acid at the MP2/junTZ Level with the Experimental Data

assignment	symmetry	ω	Cartesian	curvilinear	exp.^a^
OH str.	A′	3760	3575	3575	3583
CH_3_ a str.		3227	3083	3084	3051
CH_3_ s str.		3101	2992	2992	2944
C=O str.		1812	1782	1782	1788
CH_3_ a def.		1490	1450	1448	1430
CH_3_ s def.		1421	1380	1377	1383
OH bend		1342	1324	1322	1264
C–O str.		1206	1161	1159	1182
CH_3_ rock.		1007	988	986	989
CC str.		875	856	856	847
OCO bend		583	576	577	657
CCO bend		423	424	422	581
CH_3_ a str.	A^″^	3184	3044	3044	2996
CH_3_ a def.		1498	1440	1437	1430
CH_3_ rock.		1074	1049	1045	1048
C=O op bend		663	644	643	642
C–O torsion		552	538	537	534
CH_3_ torsion		75	85	85	93
			(−5213)	(68)	

a Ref (87).

### 5.4 Dual-Level Methods

It is well known that harmonic frequencies are more sensitive to the level of the underlying electronic Hamiltonian than higher-order force constants. The most important reason for this is the increased importance of the nuclear repulsion contribution for higher-order derivatives, with this term being always treated exactly.^88^ Furthermore, the computational cost of a full quartic force field is much higher than that of the harmonic part at the same level of theory. Finally, the whole foundation of any perturbative treatment is that the final results are more sensitive to the quality of the zero-order (harmonic) contribution than to that of the first- and second-order corrections. These considerations lead to the development of the so-called dual-level (or hybrid) methods, with the simplest one (referred to as additive approach, Add)^89^ solving the VPT2 equations employing the low-level harmonic frequencies and higher-order derivatives. Then, the results are corrected for the difference between high- and low-level harmonic frequencies. This approach is not recommended because the denominators of the perturbative contributions are evaluated by low-level harmonic frequencies, which can lead to non-negligible distortions of the results. A simple recipe for solving this problem is offered by the so-called substitution (Sub) approach^32^ in which the VPT2 equations are solved employing low-level anharmonic couplings, but high-level harmonic frequencies are used to compute the denominators.

The quality of the results obtainable by dual-level methods is analyzed in some detail for the case of uracil (see Figure 2h). Inspection of Table 8 confirms that among hybrid density functionals, the B3PW91/julDZ model represents the best compromise between accuracy and feasibility for molecules too large to be treated by state-of-the-art post-Hartree–Fock methods.^71^

**Table 8:** Comparison of Harmonic Frequencies and Curvilinear GVPT2 Fundamental Wavenumbers (in cm^–1^) of Uracil with Experimental Data

		B3		rDSD//B3			best//B3			
assignment	symm.	ω^a^	ν^a^	ω^b^	add^c^	sub^d^	ω^e^	add^f^	sub^g^	exp.^h^
N1–H str	A′	3654	3485	3661	3492	3493	3653	3484	3483	3485
N3–H str		3612	3442	3612	3442	3442	3602	3432	3428	3435
C5–H str		3265	3125	3265	3125	3125	3253	3113	3103	
C6–H str		3219	3074	3223	3078	3089	3218	3073	3069	
C=O str		1815	1785	1807	1777	1775	1790	1760	1762	1764
C4=O str		1781	1767	1774	1760	1741	1762	1748	1728	1706
C5=C6 str		1688	1652	1684	1648	1650	1678	1642	1642	1646
N1–H bend		1510	1464	1512	1466	1468	1505	1459	1460	1472
C6–H bend		1420	1387	1429	1396	1393	1427	1394	1397	1400
N3–H bend		1404	1370	1418	1384	1386	1414	1380	1381	1389
C5–H bend		1382	1347	1395	1360	1360	1394	1359	1362	1359
ring str def		1236	1204	1243	1211	1211	1248	1216	1214	1217
ring str def		1203	1177	1212	1186	1185	1205	1179	1178	1185
ring str def		1091	1073	1093	1075	1074	1084	1066	1063	1075
ring str def		992	977	997	982	980	995	980	978	980
ring str def		973	951	975	953	931	968	946	954	958
ring str def		779	755	774	750	749	773	749	766	759
ring bend def		558	555	560	557	551	545	542	541	562
ring bend def		541	532	542	533	533	541	532	536	537
ring bend def		519	512	519	512	512	517	510	510	516
C=O bend		385	384	388	387	385	387	386	374	391
C6–H op bend	A^″^	970	925	979	934	963	973	928	954	987
C5–H op bend		815	798	822	805	806	814	797	796	804
C2=O op bend		766	751	767	752	752	765	750	750	757
C4=O op bend		728	714	735	721	721	728	714	713	718
N3–H op bend		695	657	683	645	645	670	632	630	662
N1–H op bend		578	538	556	516	514	559	519	517	551
ring op def		397	384	395	382	381	388	375	385	411
ring op def		168	159	163	154	154	159	150	150	185
ring op def		151	143	146	138	138	140	132	132	
MAE			13		12	11		13	11	

a B3/julDZ.

b rDSD/junTZ.

c Hybrid model based on the additive approach employing harmonic frequencies at the rDSD/junTZ level in conjunction with anharmonic corrections at the B3/julDZ level.

d Hybrid model based on the substitution approach employing harmonic frequencies at the rDSD/junTZ level in conjunction with anharmonic corrections at the B3/julDZ level.

e Best estimate (ref (92)).

f Hybrid model based on the additive approach employing best-estimate harmonic frequencies in conjunction with anharmonic corrections at the B3/julDZ level.

g Hybrid model based on the substitution approach employing best-estimate harmonic frequencies in conjunction with anharmonic corrections at the B3/julDZ level.

h Refs (93−95).

The double hybrid rDSD functional in conjunction with a partially augmented triple-zeta basis set can be generally used to improve the harmonic part of the force field.^90,91^ In the case of uracil, B3, rDSD, and, even, coupled cluster harmonic frequencies are quite close, so that the dual-level approach does not improve the results in a significant way, but the situation is different in several other cases (e.g., glycine discussed below). From a more general perspective, the results show that the GVPT2 model is capable of providing remarkably accurate results for semi-rigid molecules plagued by significant resonances, as is the case for uracil.

**Figure 6:**
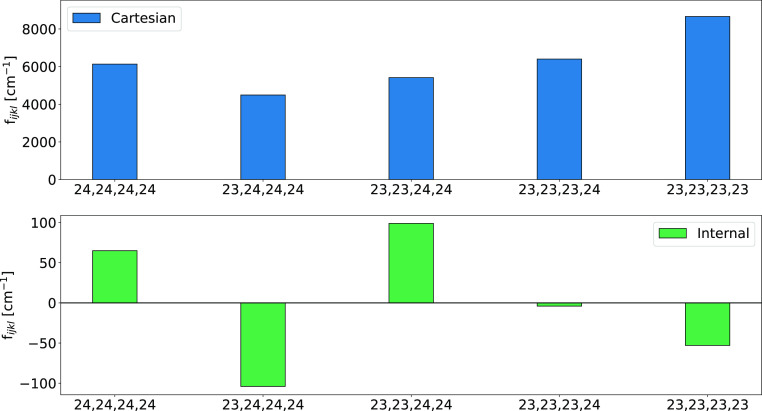
Comparison of the Cartesian (top panel) and curvilinear (bottom panel) quartic force constants of the Ip conformer of glycine involving modes 23 and 24 at the rDSD/junTZ level of theory.

As a second example, we consider the Ip conformer of glycine (see Figure 2i).^96^ The computed vibrational frequencies are compared in Table 9 with their experimental counterparts. The agreement is remarkable for all the tested computational models and, in particular, dual-level rDSD//B3 approaches reduce the average error getting closer to the results obtained at the much more expensive rDSD level. Although the results are similar for Cartesian and curvilinear coordinates, they show a very different pattern when one tries to disentangle the contribution of LAMs (ϕ and ψ torsions, modes 23 and 24 at about 200 and 90 cm^–1^). As a matter of fact, Figure 6 shows that both diagonal and off-diagonal potential anharmonic contributions are very large in Cartesian coordinates, whereas this is not the case when employing curvilinear coordinates. As a consequence, separation between SAMs and LAMs should be safer in the context of curvilinear coordinates.

**Table 9:** Comparison of the Cartesian and Curvilinear Harmonic and GVPT2 Fundamental Wavenumbers (in cm^–1^) of the Ip Conformer of Glycine with the Experimental Data

		ω		Cartesian	curvilinear				
assignment	symm.	B3^a^	rDSD^b^	rDSD^b^	B3^a^	add^c^	sub^d^	rDSD^b^	exp.
OH str	A′	3767	3766	3581	3572	3570	3571	3579	3585^e^
NH_2_ s str		3514	3521	3377	3356	3373	3366	3370	3359^f^
CH_2_ s str		3057	3068	2953	2920	2949	2938	2947	2943^f^
C=O str		1825	1817	1786	1790	1775	1783	1788	1779^g^
NH_2_ bend		1668	1682	1627	1574	1630	1616	1603	1608^f^
CH_2_ bend		1439	1472	1435	1404	1470	1437	1436	1429^f^^,^^g^
CH_2_ bend		1400	1417	1387	1362	1396	1379	1407	1405^f^
(OH + CH_2_) bend		1301	1317	1295	1271	1301	1285	1299	1297^f^
CN str + OH bend		1177	1176	1134	1140	1147	1148	1135	1136^f^^,^^g^
C=O str + OH bend		1143	1137	1102	1100	1091	1097	1103	1101^f^^,^^g^
CC str + NH_2_ bend		927	937	888	868	893	883	892	883^f^^,^^g^
CC str		832	834	808	798	803	801	811	801^f^^,^^g^
(NH_2_ + OCO) bend		634	637	633	624	630	627	636	619^f^^,^^g^
CCO(H) bend		464	467	462	451	457	454	464	458^h^
CCN bend		255	259	255	239	248	244	261	250^h^
NH_2_ a str	A^″^	3590	3599	3428	3425	3423	3414	3428	3410^f^^,^^g^
CH_2_ a str		3100	3109	2965	2957	2981	2972	2965	2969
CH_2_ bend		1376	1397	1357	1333	1377	1356	1360	1340
CH_2_ NH_2_ twist		1174	1194	1164	1145	1176	1156	1167	1166^f^
CH_2_ NH_2_ twist		913	923	911	899	918	908	913	907^f^^,^^g^
OH op bend		653	649	619	602	594	598	623	615^f^
OH op bend		509	511	495	478	483	481	499	500^g^
CN tors (ϕ)		210	217	203	151	168	161	232	204^h^
CC tors (ψ)		67	68	64	20	21	21	90	
MAE				8	16	13	11	8	

a julDZ basis set.

b junTZ basis set.

c Hybrid model based on the additive approach, employing harmonic frequencies at the rDSD/junTZ level in conjunction with anharmonic corrections at the B3/julDZ level.

d Hybrid model based on the substitution approach, employing harmonic frequencies at the rDSD/junTZ level in conjunction with anharmonic corrections at the B3/julDZ level.

e Ref (97).

f Ref (98).

g Ref (99).

h Ref (100).

Deeper insights on the role of different couplings can be obtained by comparing the results of a series of computations in which one- two- and three-mode anharmonic contributions (both potential and kinetic) are progressively added to the starting harmonic model. As already mentioned, the computational effort of electronic structure computations increases sharply with the number of different modes taken into account at the same time for potential couplings. The results collected in Table 10 show that, when employing curvilinear internal coordinates, the HCAM (harmonic coupled anharmonic modes) model has already performed a remarkable job, and inclusion of two-mode anharmonic couplings provides semi-quantitative results. These findings pave the way toward the implementation of very effective reduced-dimensionality approaches, in which only a few key anharmonic contributions are taken into account.

**Table 10:** Comparison of the Cartesian and Curvilinear Harmonic and GVPT2 Fundamental Wavenumbers (in cm^–1^) of the Ip Conformer of Glycine at the rDSD/junTZ Level of Theory Starting from Diagonal Anharmonic Couplings and Then Adding Two- and Three-Mode Couplings in a Stepwise Manner^a^

			Cartesian			curvilinear		
assignment	symm.	ω	diagonal^b^	two-mode^c^	three-mode^d^	diagonal^b^	two-mode^c^	three-mode^d^
OH str	A′	3766	3604	3527	3581	3603	3577	3579
NH_2_ s str		3521	3453	3309	3377	3449	3368	3370
CH_2_ s str		3068	3015	2914	2953	3013	2947	2947
C=O str		1817	1806	1785	1786	1806	1788	1788
NH_2_ bend		1682	1681	1732	1627	1676	1621	1603
CH_2_ bend		1472	1471	1475	1435	1470	1440	1436
CH_2_ bend		1417	1421	1391	1387	1418	1386	1407
(OH + CH_2_) bend		1317	1322	1366	1295	1315	1307	1299
CN str + OH bend		1176	1177	1162	1134	1174	1151	1135
C=O str + OH bend		1137	1149	1123	1102	1142	1111	1103
CC str + NH_2_ bend		937	946	1017	888	929	902	892
CC str		834	844	874	808	836	822	811
(NH_2_ + OCO) bend		637	639	645	633	639	638	636
CCO(H) bend		467	468	471	462	467	470	464
CCN bend		259	264	284	255	261	273	261
NH_2_ a str	A^″^	3599	3683	3297	3428	3683	3423	3428
CH_2_ a str		3109	3181	2907	2965	3181	2958	2969
CH_2_ bend		1397	1401	1378	1357	1395	1377	1340
CH_2_ NH_2_ twist		1194	1201	1188	1164	1193	1175	1167
CH_2_ NH_2_ twist		923	937	961	911	926	920	913
OH op bend		649	838	712	619	633	626	623
OH op bend		511	607	616	495	511	509	499
CN tors (ϕ)		217	1302	755	203	209	238	232
CC tors (ψ)		68	839	579	64	76	94	90
MAE			138^e^	94^e^		45^f^	8^f^	

a Kinetic and potential terms are added at the same time in the case of curvilinear coordinates.

b Calculation performed by including only diagonal terms.

c Calculation performed by including up to two-mode vibrational couplings.

d Calculation performed by including up to three-mode couplings.

e Mean absolute error computed with respect to the three-mode Cartesian-based fundamental wavenumbers.

f Mean absolute error computed with respect to the three-mode internal-based fundamental wavenumbers.

## 6 Conclusions

In this work, we have shown how the VPT2 equations for Cartesian coordinates can be extended to curvilinear internal coordinates without any additional computational bottleneck.

The results for several test cases point out the generality and robustness of the new GVPT2 engine employing curvilinear coordinates, which allows the effective treatment of medium-to-large-sized molecules for all electronic structure methods for which analytical Hessians are available. Dual-level methods combining high-level harmonic terms with lower-level anharmonic contributions further widen the range of application of the general platform.

The new development offers a number of advantages with respect to previous, ad hoc procedures. The first aspect concerns the ease of implementation since the new approach does not require any heavy modification of the codes already supporting VPT2 for asymmetric tops and Cartesian coordinates. However, the most important advantage is that the intrinsic problems of a low-order perturbative treatment based on Cartesian normal modes are strongly reduced. As a matter of fact, as clearly stated by Stanton and co-workers in connection with higher-order perturbative treatments (e.g., VPT4),^47^ VPT based on a rectilinear Hamiltonian is simply poorly suited to the problem of floppy molecular systems, and approaches such as VPT2 in curvilinear coordinates are to be preferred. While work aimed at developing more refined models for the treatment of LAMs is underway in our laboratory, we think that already the present implementation offers a number of interesting perspectives for the study of molecular systems of current scientific and technological interest.

## Supporting Information Available

Additional details about the kinetic energy operator in curvilinear coordinates, unit conversion, Wilson GF method, and resonant terms in the χ matrix (PDF)
