# Supplementary material for: Perturb-Then-Diagonalize Vibrational Engine Exploiting Curvilinear Internal Coordinates
Source: J Chem Theory Comput. 2022 Nov 2;18(12):7603–19. doi: 10.1021/acs.jctc.2c00773 (PMC9753597; doi:10.1021/acs.jctc.2c00773)
Supplement: Supplementary file 1 — ct2c00773_si_001.pdf [file ct2c00773_si_001.pdf]

# Supporting Information: Perturb-then-diagonalize vibrational engine exploiting curvilinear internal coordinates

Marco Mendolicchio,*,† Julien Bloino,‡ and Vincenzo Barone*,‡

†*Scuola Superiore Meridionale, Largo S. Marcellino 10, I-80138, Napoli (Italy)*

‡*Scuola Normale Superiore, Piazza dei Cavalieri 7, I-56126 Pisa (Italy)*

E-mail: marco.mendolicchio-ssm@unina.it; vincenzo.barone@sns.it

# Contents

**S1 Kinetic energy operator in curvilinear coordinates** **S-3**

**S2 Wilson GF method** **S-5**

**S3 Conversion of energy units and Wilson G matrix derivatives** **S-8**

S3.1 Kinetic-energy derivatives . . . . . . . . . . . . . . . . . . . . . . . . . . . . S-9

S3.2 Potential-energy derivatives . . . . . . . . . . . . . . . . . . . . . . . . . . . S-10

**S4 $\chi$ matrix for Cartesian normal modes** **S-11**

**S5 Resonant terms in the $\chi$ matrix** **S-12**

S5.1 Potential term . . . . . . . . . . . . . . . . . . . . . . . . . . . . . . . . . . S-13

S5.2 Kinetic term . . . . . . . . . . . . . . . . . . . . . . . . . . . . . . . . . . . S-13

S5.3 Cross term . . . . . . . . . . . . . . . . . . . . . . . . . . . . . . . . . . . . S-14

**References** **S-14**

## S1 Kinetic energy operator in curvilinear coordinates

In the following, the mathematical steps to convert the standard equation of the internal-based kinetic energy operator $\mathcal{T}$ into a form where different contributions are separated, are shown.

For readability, the initial expression[S1] is recalled here,

$$\mathcal{T} = -\frac{\hbar^2}{2}\sum_{i=1}^{N}\sum_{j=1}^{N}\tilde{\mathsf{G}}^{1/4}\frac{\partial}{\partial s_i}\tilde{\mathsf{G}}^{-1/2}\mathsf{G}_{ij}\frac{\partial}{\partial s_j}\tilde{\mathsf{G}}^{1/4} \tag{S1}$$

where $\boldsymbol{s} = \{1, \ldots, s_n\}$ is a complete and non-redundant set of internal coordinates and $\tilde{\mathsf{G}}$ is the determinant of the Wilson $\mathbf{G}$ matrix.

In order to rewrite Eq. S1, the application of the differential operator $\mathcal{T}$ to a generic function $\Phi(\boldsymbol{s})$ must be explored:

$$
\begin{aligned}
\mathcal{T}\Phi &= -\frac{\hbar^2}{2}\sum_{i=1}^{N}\sum_{j=1}^{N}\tilde{\mathsf{G}}^{1/4}\frac{\partial}{\partial s_i}\tilde{\mathsf{G}}^{-1/2}\mathsf{G}_{ij}\frac{\partial}{\partial s_j}\tilde{\mathsf{G}}^{1/4}\Phi \\
&= -\frac{\hbar^2}{2}\sum_{i=1}^{N}\sum_{j=1}^{N}\tilde{\mathsf{G}}^{1/4}\frac{\partial}{\partial s_i}\tilde{\mathsf{G}}^{-1/2}\mathsf{G}_{ij}\left(\frac{1}{4}\tilde{\mathsf{G}}^{-3/4}\frac{\partial\tilde{\mathsf{G}}}{\partial s_j}\Phi + \tilde{\mathsf{G}}^{1/4}\frac{\partial\Phi}{\partial s_j}\right) \\
&= -\frac{\hbar^2}{2}\sum_{i=1}^{N}\sum_{j=1}^{N}\tilde{\mathsf{G}}^{1/4}\frac{\partial}{\partial s_i}\left(\frac{1}{4}\tilde{\mathsf{G}}^{-5/4}\mathsf{G}_{ij}\frac{\partial\tilde{\mathsf{G}}}{\partial s_j}\Phi + \tilde{\mathsf{G}}^{-1/4}\mathsf{G}_{ij}\frac{\partial\Phi}{\partial s_j}\right) \\
&= -\frac{\hbar^2}{8}\sum_{i=1}^{N}\sum_{j=1}^{N}\tilde{\mathsf{G}}^{1/4}\frac{\partial}{\partial s_i}\left(\tilde{\mathsf{G}}^{-5/4}\mathsf{G}_{ij}\frac{\partial\tilde{\mathsf{G}}}{\partial s_j}\Phi + 4\tilde{\mathsf{G}}^{-1/4}\mathsf{G}_{ij}\frac{\partial\Phi}{\partial s_j}\right) \\
&= -\frac{\hbar^2}{8}\sum_{i=1}^{N}\sum_{j=1}^{N}\tilde{\mathsf{G}}^{1/4}\left(-\frac{5}{4}\tilde{\mathsf{G}}^{-9/4}\frac{\partial\tilde{\mathsf{G}}}{\partial s_i}\mathsf{G}_{ij}\frac{\partial\tilde{\mathsf{G}}}{\partial s_j}\Phi + \tilde{\mathsf{G}}^{-5/4}\frac{\partial\mathsf{G}_{ij}}{\partial s_i}\frac{\partial\tilde{\mathsf{G}}}{\partial s_j}\Phi\right. \\
&\quad +\tilde{\mathsf{G}}^{-5/4}\mathsf{G}_{ij}\frac{\partial^2\tilde{\mathsf{G}}}{\partial s_i\partial s_j}\Phi + \tilde{\mathsf{G}}^{-5/4}\mathsf{G}_{ij}\frac{\partial\tilde{\mathsf{G}}}{\partial s_j}\frac{\partial\Phi}{\partial s_i} - \tilde{\mathsf{G}}^{-5/4}\frac{\partial\tilde{\mathsf{G}}}{\partial s_i}\mathsf{G}_{ij}\frac{\partial\Phi}{\partial s_j} \\
&\quad \left.+4\tilde{\mathsf{G}}^{-1/4}\frac{\partial\mathsf{G}_{ij}}{\partial s_i}\frac{\partial\Phi}{\partial s_j} + 4\tilde{\mathsf{G}}^{-1/4}\mathsf{G}_{ij}\frac{\partial^2\Phi}{\partial s_i\partial s_j}\right) \\
&= -\frac{\hbar^2}{8}\sum_{i=1}^{N}\sum_{j=1}^{N}\left(-\frac{5}{4\tilde{\mathsf{G}}^2}\frac{\partial\tilde{\mathsf{G}}}{\partial s_i}\mathsf{G}_{ij}\frac{\partial\tilde{\mathsf{G}}}{\partial s_j}\Phi + \frac{1}{\tilde{\mathsf{G}}}\frac{\partial\mathsf{G}_{ij}}{\partial s_i}\frac{\partial\tilde{\mathsf{G}}}{\partial s_j}\Phi + \frac{\mathsf{G}_{ij}}{\tilde{\mathsf{G}}}\frac{\partial^2\tilde{\mathsf{G}}}{\partial s_i\partial s_j}\Phi\right. \\
&\quad \left.+\frac{\mathsf{G}_{ij}}{\tilde{\mathsf{G}}}\frac{\partial\tilde{\mathsf{G}}}{\partial s_j}\frac{\partial\Phi}{\partial s_i} - \frac{1}{\tilde{\mathsf{G}}}\frac{\partial\tilde{\mathsf{G}}}{\partial s_i}\mathsf{G}_{ij}\frac{\partial\Phi}{\partial s_j} + 4\frac{\partial\mathsf{G}_{ij}}{\partial s_i}\frac{\partial\Phi}{\partial s_j} + 4\mathsf{G}_{ij}\frac{\partial^2\Phi}{\partial s_i\partial s_j}\right) \\
&= -\frac{\hbar^2}{8}\sum_{i=1}^{N}\sum_{j=1}^{N}\left(-\frac{5}{4}\mathsf{G}_{ij}\frac{\partial\ln\tilde{\mathsf{G}}}{\partial s_i}\frac{\partial\ln\tilde{\mathsf{G}}}{\partial s_j} + \frac{\partial\mathsf{G}_{ij}}{\partial s_i}\frac{\partial\ln\tilde{\mathsf{G}}}{\partial s_j} + \frac{\mathsf{G}_{ij}}{\tilde{\mathsf{G}}}\frac{\partial^2\tilde{\mathsf{G}}}{\partial s_i\partial s_j}\right. \\
&\quad \left.+\mathsf{G}_{ij}\frac{\partial\ln\tilde{\mathsf{G}}}{\partial s_j}\frac{\partial}{\partial s_i} - \mathsf{G}_{ij}\frac{\partial\ln\tilde{\mathsf{G}}}{\partial s_i}\frac{\partial}{\partial s_j} + 4\frac{\partial\mathsf{G}_{ij}}{\partial s_i}\frac{\partial}{\partial s_j} + 4\mathsf{G}_{ij}\frac{\partial^2}{\partial s_i\partial s_j}\right)\Phi
\end{aligned}
\tag{S2}
$$

Now it is possible to notice that the fourth and fifth terms within parenthesis can be simplified,

$$
\begin{aligned}
\mathcal{T} = &-\frac{\hbar^2}{2}\sum_{i=1}^{N}\sum_{j=1}^{N}\mathsf{G}_{ij}\frac{\partial^2}{\partial s_i\partial s_j} - \frac{\hbar^2}{2}\sum_{i=1}^{N}\sum_{j=1}^{N}\frac{\partial\mathsf{G}_{ij}}{\partial s_i}\frac{\partial}{\partial s_j} \\
&+\frac{\hbar^2}{8}\sum_{i=1}^{N}\sum_{j=1}^{N}\left(\frac{5}{4}\mathsf{G}_{ij}\frac{\partial\ln\tilde{\mathsf{G}}}{\partial s_i}\frac{\partial\ln\tilde{\mathsf{G}}}{\partial s_j} - \frac{\mathsf{G}_{ij}}{\tilde{\mathsf{G}}}\frac{\partial^2\tilde{\mathsf{G}}}{\partial s_i\partial s_j} - \frac{\partial\mathsf{G}_{ij}}{\partial s_i}\frac{\partial\ln\tilde{\mathsf{G}}}{\partial s_j}\right)
\end{aligned}
\tag{S3}
$$

and upon rearrangement, the kinetic energy operator can be written as

$$\begin{aligned}\mathcal{T} = &-\frac{\hbar^2}{2}\sum_{i=1}^{N}\sum_{j=1}^{N}\frac{\partial^2}{\partial s_i \partial s_j} - \frac{\hbar^2}{2}\sum_{i=1}^{N}\sum_{j=1}^{N}\frac{\partial \mathsf{G}_{ij}}{\partial s_i}\frac{\partial}{\partial s_j} \\ &+ \frac{\hbar^2}{32}\sum_{i=1}^{N}\sum_{j=1}^{N}\left[\mathsf{G}_{ij}\frac{\partial \tilde{\mathsf{G}}}{\partial s_i}\frac{\partial \tilde{\mathsf{G}}}{\partial s_j} - 4\frac{\partial}{\partial s_i}\left(\mathsf{G}_{ij}\frac{\partial \tilde{\mathsf{G}}}{\partial s_j}\right)\right]\end{aligned} \tag{S4}$$

Finally, a further algebraic manipulations yields the following, reference expression,

$$\mathcal{T} = -\frac{\hbar^2}{2}\sum_{i=1}^{N}\sum_{j=1}^{N}\frac{\partial}{\partial s_i}\mathsf{G}_{ij}\frac{\partial}{\partial s_j} + \mathcal{V}_{\mathsf{g}} \tag{S5}$$

where $\mathcal{V}'$ is known as extra-potential term, whose expression is reported below:

$$\mathcal{V}_{\mathsf{g}} = \frac{\hbar^2}{32}\sum_{i=1}^{N}\sum_{j=1}^{N}\left[\mathsf{G}_{ij}\frac{\partial \tilde{\mathsf{G}}}{\partial s_i}\frac{\partial \tilde{\mathsf{G}}}{\partial s_j} - 4\frac{\partial}{\partial s_i}\left(\mathsf{G}_{ij}\frac{\partial \tilde{\mathsf{G}}}{\partial s_j}\right)\right] \tag{S6}$$

# S2 Wilson GF method

In analogy with the treatment in terms of Cartesian-based normal coordinates (CNCs), the harmonic approximation of vibrations can be employed, through the so-called Wilson GF method.[S2] Within this method, the **G** matrix is approximated with its value at the reference geometry (usually the equilibrium one). As a result, the dependence of the **G** matrix on the internal coordinates is neglected, as well as that of its determinant $\tilde{\mathsf{G}}$, so that,

$$\mathcal{T} = -\frac{\hbar^2}{2}\sum_{i=1}^{N}\sum_{j=1}^{N}G_{ij}^{\text{eq}}\frac{\partial^2}{\partial s_i \partial s_j} \tag{S7}$$

where the superscript "eq" indicates that the elements $\mathsf{G}_{ij}$ are evaluated at the equilibrium geometry, and from here on will be omitted.

The treatment of potential energy is identical to that employed in terms of CNCs, the only difference being the set of coordinates used for the Taylor-series expansion,

$$\mathcal{V} = \mathcal{V}^{\text{eq}} + \sum_{i=1}^{N} \left(\frac{\partial \mathcal{V}}{\partial s_i}\right)_{\text{eq}} s_i + \frac{1}{2}\sum_{i=1}^{N}\sum_{j=1}^{N} \left(\frac{\partial^2 \mathcal{V}}{\partial s_i \partial s_j}\right)_{\text{eq}} s_i s_j + \mathcal{O}(|\boldsymbol{s}|^2) \tag{S8}$$

By assuming the condition of stationary point and shifting the origin of the PES to zero, the above equation truncated to the second order can be written as,

$$\mathcal{V} = \frac{1}{2}\sum_{i=1}^{N}\sum_{j=1}^{N} F_{ij} s_i s_j \tag{S9}$$

where $\mathbf{F}$ is the Hessian matrix of the potential energy with respect to the internal coordinates. While the $\mathbf{G}$ matrix is easily obtainable on the basis of structural parameters (namely the molecular geometry and atomic masses), $\mathbf{F}$ can be calculated from the Cartesian Hessian matrix $\mathbf{H}_{\text{x}}$. In the most general case, also accounting for non-equilibrium geometries, its expression is,[S3–S6]

$$\mathbf{F} = \{\mathbf{B}^{\dagger}\}^{\text{T}}[\mathbf{H}_{\boldsymbol{x}} - \boldsymbol{g}_s \mathbf{B}']\mathbf{B}^{\dagger} \tag{S10}$$

where $\boldsymbol{g}_s$ is the gradient of the potential expressed in internal coordinates, which can be calculated from its Cartesian counterpart $\boldsymbol{g}_{\boldsymbol{x}}$,[S3,S4]

$$\boldsymbol{g}_s = \{\mathbf{B}^{\dagger}\}^{\text{T}} \boldsymbol{g}_x \tag{S11}$$

while $\mathbf{B}^{\dagger}$ is the Moore-Penrose pseudo-inverse of $\mathbf{B}$, defined as[S4]

$$\mathbf{B}^{\dagger} = (\mathbf{B}\mathbf{U}\mathbf{B}^{\text{T}})^{-1}\mathbf{B}^{\text{T}}\mathbf{U} \tag{S12}$$

$\mathbf{U}$ is a $(3N_a \times 3N_a)$ arbitrary matrix, with $N_a$ being the number of atoms. It is noteworthy that the contribution of translations and rotations can be factored out through the application of the projection matrix $\overline{\mathbf{P}} = \mathbf{B}^{\dagger}\mathbf{B}$. More specifically, $\boldsymbol{g}_s$ and $\mathbf{F}$ can be obtained as,[S4,S7]

$$
\begin{aligned}
\boldsymbol{g}_s &= \{\mathbf{B}^\dagger\}^{\mathrm{T}}\, \overline{\mathbf{P}}\, \boldsymbol{g}_x \\
\mathbf{F} &= \{\mathbf{B}^\dagger\}^{\mathrm{T}}\, \overline{\mathbf{P}}\, [\mathbf{H}_x - \boldsymbol{g}_s \mathbf{B}']\, \overline{\mathbf{P}}\, \mathbf{B}^\dagger
\end{aligned}
\tag{S13}
$$

Once this procedure has been carried out, the harmonic vibrational Hamiltonian $\mathcal{H}^{(0)}$ can be defined as,

$$
\mathcal{H}^{(0)} = \frac{1}{2} \sum_{i=1}^{N} \sum_{j=1}^{N} \left( \mathsf{G}_{ij} P_i^s P_j^s + F_{ij} s_i s_j \right) \tag{S14}
$$

where $P_i^s = -i\hbar\partial/\partial s_i$.

At variance with the expansion in terms of mass-weighted Cartesian displacements, the kinetic energy is not diagonal, the different coordinates being coupled by the **G** matrix. As a result, a set of coordinates diagonalizing both **F** and **G**, must be defined. For this purpose, a set of normal coordinates **Q** can be introduced,

$$
\boldsymbol{s} = \mathbf{L}\, \mathbf{Q} \tag{S15}
$$

By inserting Eq. S15 in Eq. S14, and recasting the latter in a matrix form, we obtain the following expression,

$$
\mathcal{H}^{(0)} = \frac{1}{2} \left[ \boldsymbol{P}^{\mathrm{T}} \mathbf{L}^{-1} \mathbf{G} (\mathbf{L}^{-1})^{\mathrm{T}} \boldsymbol{P} + \boldsymbol{Q}^{T} \mathbf{L}^{\mathrm{T}} \mathbf{F} \mathbf{L} \boldsymbol{Q} \right] \tag{S16}
$$

**L** is defined so that in this basis the **F** matrix becomes diagonal ($\boldsymbol{\Lambda}$), while **G** is equal to the identity matrix. This corresponds to the resolution of the following equation,

$$
\mathbf{GFL} = \mathbf{L}\boldsymbol{\Lambda} \tag{S17}
$$

In other words, the calculation of the harmonic frequencies and internal-based normal coordinates (INCs) can be performed through the diagonalization of the **GF** matrix product.

It should be noted that for curvilinear coordinates the matrix to be diagonalized is not symmetric, implying that the normal coordinates do not form an orthogonal basis. However, as demonstrated by Myazawa,[S8] Eq. S17 can be recast in a symmetric form characterized by the same eigenvalues,

$$(\mathbf{G}^{1/2}\mathbf{F}\mathbf{G}^{1/2})(\mathbf{G}^{-1/2}\mathbf{L}) = (\mathbf{G}^{-1/2}\mathbf{L})\Lambda \tag{S18}$$

where the columns of the matrix $\mathbf{G}^{-1/2}\mathbf{L}$ are the eigenvectors of the symmetric matrix $\mathbf{G}^{1/2}\mathbf{F}\mathbf{G}^{1/2}$, and thus are orthogonal.

It is important to point out that the harmonic Hamiltonian $\mathcal{H}^{(0)}$ introduced in Eq. S14, can be converted to the basis of the dimensionless normal coordinates $\boldsymbol{q}$, leading to the following expression,

$$\mathcal{H}^{(0)} = \frac{1}{2}\sum_{i=1}^{N}\omega_i(q_i^2 + p_i^2) \tag{S19}$$

where $p_i = -i\partial/\partial q_i$. Since the expression of $\mathcal{H}^{(0)}$ through dimensionless units is equivalent to that obtained in the Cartesian-based formulation, the eigenvalues and eigenfunctions of $\mathcal{H}^{(0)}$ are still given by Hermite polynomials multiplied by gaussian functions. Moreover, the second quantization formalism can be employed in the derivation of the equations stemming from the perturbative treatment.

# S3 Conversion of energy units and Wilson G matrix derivatives

In this section, the expressions required to convert both energy and Wilson $\mathbf{G}$ matrix derivatives to wavenumbers is shortly discussed. For the purpose, it is necessary to introduce the so-called dimensionless normal coordinates $q_i$ ($i = 1, \ldots, N$, with $N$ being the number of normal modes) in terms of the mass-weighted normal coordinates $Q_i$ (in $\sqrt{\text{amu}}$·Bohr),

$$q_i = \sqrt{\gamma_i} Q_i \tag{S20}$$

where $\gamma_i = 2\pi c\omega_i/\hbar$, $c$ is the light speed (in cm/s), $\omega_i$ is the harmonic wavenumber related to the $i$-th mode (in $\mathrm{cm}^{-1}$) and $\hbar$ is the reduced Plank constant (in amu·Bohr$^2$s$^{-1}$). The conjugate moment of $q_i$ is $p_i = -i\partial/\partial q_i$.

## S3.1 Kinetic-energy derivatives

Let us consider a generic element $\mathsf{G}_{ij}$ of the Wilson $\mathbf{G}$ matrix and let us define its derivative with respect to the mass-weighted normal coordinates

$$\mathsf{G}_{ij,kl,\ldots} = \frac{\partial^n \mathsf{G}_{ij}}{\partial Q_k \partial Q_l \ldots} \tag{S21}$$

Let us now introduce the $\mathbf{g}$ matrix, obtained by converting the $\mathbf{G}$ matrix to wavenumbers, and its derivative with respect to the dimensionless normal coordinates:

$$\mathsf{g}_{ij,kl\ldots} = \frac{\partial^n \mathsf{G}_{ij}}{\partial q_k \partial q_l \ldots} \tag{S22}$$

The transformation between the two derivatives is given by

$$\mathsf{g}_{ij\ ,\ kl\ldots} = \frac{\hbar}{2\pi c}\sqrt{\frac{\gamma_i\gamma_j}{\gamma_k\gamma_l\ldots}}\,\mathsf{G}_{ij\ldots\ ,\ kl\ldots} \tag{S23}$$

By considering the zero-th order term and the first- and second-order derivatives of Eq. S23, corresponding to the contributions appearing in the perturbative expansion of the kinetic energy operator up to the second-order, the following expression arises

$$\begin{aligned} \mathsf{g}_{ij} &= \sqrt{\omega_i \omega_j} \mathsf{G}_{ij} \\ \mathsf{g}_{ij,k} &= \sqrt{\frac{\hbar}{2\pi c}} \sqrt{\frac{\omega_i \omega_j}{\omega_k}} \mathsf{G}_{ij,k} \\ \mathsf{g}_{ij,kl} &= \frac{\hbar}{2\pi c} \sqrt{\frac{\omega_i \omega_j}{\omega_k \omega_l}} \mathsf{G}_{ij,kl} \end{aligned} \tag{S24}$$

Furthermore, the Wilson **G** matrix is equal to the identity matrix at the equilibrium geometry:

$$\mathsf{g}_{ij}^{\mathrm{eq}} = \omega_i \delta_{ij} \tag{S25}$$

## S3.2 Potential-energy derivatives

Let us consider a generic anharmonic force constant with respect to the mass-weighted normal coordinates,

$$\mathsf{F}_{ijk,\ldots} = \left( \frac{\partial^n V}{\partial Q_i \partial Q_j \partial Q_k} \right)_{\mathrm{eq}} \tag{S26}$$

The corresponding counterpart expressed in wavenumbers is

$$\mathsf{f}_{ijk,\ldots} = \left( \frac{\partial^n V}{\partial q_i \partial q_j \partial q_k} \right)_{\mathrm{eq}} \tag{S27}$$

The transformation between the two derivatives is given by

$$\mathsf{f}_{ijk\ldots} = \frac{1}{hc} \frac{1}{\sqrt{\gamma_i \gamma_j \gamma_k \ldots}} \mathsf{F}_{ijk\ldots} \tag{S28}$$

By considering the second-, third- and fourth-order derivatives of Eq. S28, corresponding to the contributions appearing in the perturbative expansion of the potential energy up to the second-order, the following expressions are obtained:

$$
\begin{aligned}
\mathsf{f}_{ij} &= \frac{1}{4\pi^2 c^2}\frac{\mathsf{F}_{ij}}{\sqrt{\omega_i\omega_j}} \\
\mathsf{f}_{ijk} &= \frac{1}{4\pi^2 c^2}\sqrt{\frac{\hbar}{2\pi c}}\frac{\mathsf{F}_{ijk}}{\sqrt{\omega_i\omega_j\omega_k}} \\
\mathsf{f}_{ijkl} &= \frac{\hbar}{8\pi^3 c^3}\frac{\mathsf{F}_{ijkl}}{\sqrt{\omega_i\omega_j\omega_k\omega_l}}
\end{aligned}
\tag{S29}
$$

Let us stress that $F_{ij} = 4\pi^2 c^2 \omega_i^2 \delta_{ij}$ at the equilibrium geometry, so that

$$
\mathsf{f}_{ij}^{\mathrm{eq}} = \omega_i \delta_{ij} \tag{S30}
$$

# S4 $\chi$ matrix for Cartesian normal modes

The anharmonic $\boldsymbol{\chi}$ matrix in the Cartesian-based formulation of VPT2 is

$$
16\chi_{ii} = \mathsf{f}_{iiii} - \frac{5\mathsf{f}_{iii}{}^2}{3\omega_i} - \sum_{\substack{j=1\\(j\neq i)}}^{N} \frac{\left(8\omega_i{}^2 - 3\omega_j{}^2\right)\mathsf{f}_{iij}{}^2}{\omega_j\left(4\omega_i{}^2 - \omega_j{}^2\right)} \tag{S31}
$$

$$
\begin{aligned}
4\chi_{ij} = \mathsf{f}_{iijj} &- \frac{2\omega_i \mathsf{f}_{iij}{}^2}{\left(4\omega_i{}^2 - \omega_j{}^2\right)} - \frac{2\omega_j \mathsf{f}_{ijj}{}^2}{\left(4\omega_j{}^2 - \omega_i{}^2\right)} - \frac{\mathsf{f}_{iii}\mathsf{f}_{ijj}}{\omega_i} - \frac{\mathsf{f}_{jjj}\mathsf{f}_{iij}}{\omega_j} \\
&+ \sum_{\substack{k=1\\(k\neq i,j)}}^{N} \left[\frac{2\omega_k\left(\omega_i{}^2 + \omega_j{}^2 - \omega_k{}^2\right)\mathsf{f}_{ijk}{}^2}{(\omega_i+\omega_j+\omega_k)(\omega_i-\omega_j-\omega_k)(\omega_j-\omega_i-\omega_k)(\omega_k-\omega_i-\omega_j)} - \frac{\mathsf{f}_{iik}\mathsf{f}_{jjk}}{\omega_k}\right] \\
&+ \frac{4\left(\omega_i{}^2 + \omega_j{}^2\right)}{\omega_i\omega_j} \sum_{\tau=x,y,z} B_\tau^{\mathrm{eq}}\{\zeta_{ij,\tau}\}^2
\end{aligned}
\tag{S32}
$$

The expressions reported above can be rearranged in order to isolate the potentially resonant contributions:

$$16\chi_{ii} = \mathsf{f}_{iiii} - \frac{5\mathsf{f}_{iii}{}^2}{3\omega_i} - \sum_{\substack{j=1 \\ (j\neq i)}}^{N} \frac{\mathsf{f}_{iij}^2}{2} \left[\frac{4}{\omega_j} + \frac{1}{2\omega_i+\omega_j} - \frac{1}{2\omega_i-\omega_j}\right] \tag{S33}$$

$$\begin{aligned} 4\chi_{ij} = \mathsf{f}_{iijj} &- \frac{\mathsf{f}_{iij}{}^2}{2}\left[\frac{1}{2\omega_i+\omega_j} + \frac{1}{2\omega_i-\omega_j}\right] - \frac{\mathsf{f}_{ijj}{}^2}{2}\left[\frac{1}{2\omega_j+\omega_i} + \frac{1}{2\omega_j-\omega_i}\right] \\ &- \frac{\mathsf{f}_{iii}\mathsf{f}_{ijj}}{\omega_i} - \frac{\mathsf{f}_{jjj}\mathsf{f}_{iij}}{\omega_j} + \sum_{\substack{k=1 \\ (k\neq i,j)}}^{N} \left\{\frac{\mathsf{f}_{ijk}{}^2}{2}\left[\frac{1}{\omega_i+\omega_j+\omega_k} + \frac{1}{\omega_i-\omega_j+\omega_k} - \frac{1}{\omega_i+\omega_j-\omega_k}\right.\right. \\ &\left.\left. - \frac{1}{\omega_i-\omega_j-\omega_k}\right] - \frac{\mathsf{f}_{iik}\mathsf{f}_{jjk}}{\omega_k}\right\} + 4\left(\frac{\omega_i}{\omega_j} + \frac{\omega_j}{\omega_i}\right)\sum_{\tau=x,y,z} B_\tau^{\mathrm{eq}}\{\zeta_{ij,\tau}\}^2 \end{aligned} \tag{S34}$$

# S5 Resonant terms in the $\chi$ matrix

In this section, the potential ($\boldsymbol{\chi}^{\mathcal{V}}$), kinetic ($\boldsymbol{\chi}^{\mathcal{T}}$) and cross ($\boldsymbol{\chi}^{\times}$) contributions to the internal-based formulation of the $\boldsymbol{\chi}$ matrix, whose potentially resonant contributions have been isolated, are reported.

Such expressions are obtained starting from Eqs. 34 to 39 and applying the following identities,

$$\begin{aligned} \frac{1}{4\omega_i^2-\omega_j^2} &= \frac{1}{4\omega_i}\left[\frac{1}{2\omega_i+\omega_j} + \frac{1}{2\omega_i-\omega_j}\right] = \frac{1}{2\omega_j}\left[\frac{1}{2\omega_i-\omega_j} - \frac{1}{2\omega_i+\omega_j}\right] \\ \frac{1}{\Delta_{ijk}} &= \frac{1}{8\omega_i\omega_j\omega_k}\left[\frac{1}{\omega_i+\omega_j+\omega_k} - \frac{1}{\omega_i-\omega_j+\omega_k} - \frac{1}{\omega_i+\omega_j-\omega_k} + \frac{1}{\omega_i-\omega_j-\omega_k}\right] \\ \frac{\omega_k^2-\omega_i^2-\omega_j^2}{\Delta_{ijk}} &= \frac{1}{4\omega_k}\left[\frac{1}{\omega_i+\omega_j+\omega_k} + \frac{1}{\omega_i-\omega_j+\omega_k} - \frac{1}{\omega_i+\omega_j-\omega_k} - \frac{1}{\omega_i-\omega_j-\omega_k}\right] \\ \frac{\omega_i^2-\omega_j^2-\omega_k^2}{\Delta_{ijk}} &= \frac{1}{4\omega_i}\left[\frac{1}{\omega_i+\omega_j+\omega_k} + \frac{1}{\omega_i-\omega_j+\omega_k} + \frac{1}{\omega_i-\omega_j-\omega_k} + \frac{1}{\omega_i+\omega_j-\omega_k}\right] \\ \frac{\omega_j^2-\omega_i^2-\omega_k^2}{\Delta_{ijk}} &= \frac{1}{4\omega_j}\left[\frac{1}{\omega_i+\omega_j+\omega_k} - \frac{1}{\omega_i-\omega_j+\omega_k} + \frac{1}{\omega_i+\omega_j-\omega_k} - \frac{1}{\omega_i-\omega_j-\omega_k}\right] \end{aligned} \tag{S35}$$

where

$$\Delta_{ijk} = (\omega_i + \omega_j + \omega_k)(\omega_i - \omega_j - \omega_k)(\omega_i - \omega_j + \omega_k)(\omega_i + \omega_j - \omega_k)$$

## S5.1 Potential term

The partial fraction decomposition of the potential term is given by,

$$16\chi_{ii}^{\mathcal{V}} = \mathsf{f}_{iiii} - \frac{5\mathsf{f}_{iii}{}^2}{3\omega_i} - \sum_{\substack{j=1 \\ (j\neq i)}} \frac{\mathsf{f}_{iij}{}^2}{2}\left[\frac{4}{\omega_j} + \frac{1}{2\omega_i + \omega_j} - \frac{1}{2\omega_i - \omega_j}\right] \tag{S36}$$

$$\chi_{ij}^{\mathcal{V}} = \mathsf{f}_{iijj} - \frac{\mathsf{f}_{iij}{}^2}{2}\left[\frac{1}{2\omega_i + \omega_j} + \frac{1}{2\omega_i - \omega_j}\right] - \frac{\mathsf{f}_{ijj}{}^2}{2}\left[\frac{1}{2\omega_j + \omega_i} + \frac{1}{2\omega_j - \omega_i}\right] \tag{S37}$$
$$- \frac{\mathsf{f}_{iii}\mathsf{f}_{ijj}}{4\omega_i} - \frac{\mathsf{f}_{iij}\mathsf{f}_{jjj}}{4\omega_j} - \frac{1}{2}\sum_{\substack{k=1 \\ (k\neq i,j)}}\left[\frac{\mathsf{f}_{ijk}{}^2}{\omega_i + \omega_j + \omega_k} + \frac{\mathsf{f}_{ijk}{}^2}{\omega_i - \omega_j + \omega_k} - \frac{\mathsf{f}_{ijk}{}^2}{\omega_i + \omega_j - \omega_k}\right.$$
$$\left. - \frac{\mathsf{f}_{ijk}{}^2}{\omega_i - \omega_j - \omega_k} + \frac{2\mathsf{f}_{iik}\mathsf{f}_{jjk}}{\omega_k}\right]$$

## S5.2 Kinetic term

The partial fraction decomposition of the kinetic term is given by,

$$16\chi_{ii}^{\mathcal{T}} = 2\mathsf{g}_{ii,ii} - \frac{3\mathsf{g}_{ii,i}^2}{\omega_i} - \frac{1}{2}\sum_{\substack{j=1 \\ (j\neq i)}}\left[\frac{4\mathsf{g}_{ii,j}{}^2}{\omega_j} + \frac{\mathsf{s}_{iij}{}^2}{2\omega_i + \omega_j} - \frac{\mathsf{r}_{iij}{}^2}{2\omega_i - \omega_j}\right] \tag{S38}$$

$$4\chi_{ij}^{\mathcal{T}} = \mathsf{g}_{ii,jj} + \mathsf{g}_{jj,ii} - \frac{1}{2}\left[\frac{2\mathsf{g}_{ii,i}\mathsf{g}_{jj,i}}{\omega_i} + \frac{\mathsf{s}_{iij}{}^2}{2\omega_i + \omega_j} + \frac{\mathsf{r}_{iij}{}^2}{2\omega_i - \omega_j}\right]$$
$$- \frac{1}{2}\left[\frac{2\mathsf{g}_{ii,j}\mathsf{g}_{jj,j}}{\omega_j} + \frac{\mathsf{s}_{jji}{}^2}{2\omega_j + \omega_i} + \frac{\mathsf{r}_{jji}{}^2}{2\omega_j - \omega_i}\right] \tag{S39}$$
$$- \frac{1}{2}\sum_{\substack{k=1 \\ (k\neq i,j)}}\left[\frac{\mathsf{s}_{ijk}{}^2}{\omega_i + \omega_j + \omega_k} - \frac{\mathsf{r}_{ijk}{}^2}{\omega_i + \omega_j - \omega_k} + \frac{\mathsf{r}_{ikj}{}^2}{\omega_i - \omega_j + \omega_k} - \frac{\mathsf{r}_{jki}{}^2}{\omega_i - \omega_j - \omega_k} + \frac{2\mathsf{g}_{ii,k}\mathsf{g}_{jj,k}}{\omega_k}\right]$$

where

$$\mathsf{s}_{ijk} = \mathsf{g}_{ij,k} + \mathsf{g}_{ik,j} + \mathsf{g}_{jk,i}$$
$$\mathsf{r}_{ijk} = \mathsf{g}_{ij,k} - \mathsf{g}_{ik,j} - \mathsf{g}_{jk,i} \tag{S40}$$

## S5.3 Cross term

The partial fraction decomposition of the cross term is given by,

$$4\chi_{ii}^{\times} = -2\mathsf{g}_{ii,i}\mathsf{f}_{iii} + \sum_{\substack{j=1\\(j\neq i)}} \left[ \frac{2\mathsf{f}_{\mathsf{iij}}\mathsf{s}_{iij}}{2\omega_i + \omega_j} + \frac{2\mathsf{f}_{\mathsf{iij}}\mathsf{r}_{iij}}{2\omega_i - \omega_j} - \frac{4\mathsf{f}_{\mathsf{iij}}\mathsf{g}_{ii,j}}{\omega_j} \right] \tag{S41}$$

$$\begin{aligned} 4\chi_{ij}^{\times} = & -\frac{\mathsf{g}_{ii,i}\mathsf{f}_{ijj} + \mathsf{g}_{jj,i}\mathsf{f}_{iii}}{\omega_i} - \frac{\mathsf{g}_{jj,j}\mathsf{f}_{iij} + \mathsf{g}_{ii,j}\mathsf{f}_{jjj}}{\omega_j} \\ & + \mathsf{f}_{iij}\left[\frac{\mathsf{s}_{iij}}{2\omega_i + \omega_j} + \frac{\mathsf{r}_{iij}}{2\omega_i - \omega_j}\right] + \mathsf{f}_{ijj}\left[\frac{\mathsf{s}_{jji}}{2\omega_j + \omega_i} + \frac{\mathsf{r}_{jji}}{2\omega_j - \omega_i}\right] \\ & + \sum_{\substack{k=1\\(k\neq i,j)}} \left[\frac{\mathsf{f}_{ijk}\mathsf{s}_{ijk}}{\omega_i + \omega_j + \omega_k} - \frac{\mathsf{f}_{ijk}\mathsf{r}_{ijk}}{\omega_i + \omega_j - \omega_k} + \frac{\mathsf{f}_{ijk}\mathsf{r}_{ikj}}{\omega_i - \omega_j + \omega_k} - \frac{\mathsf{f}_{ijk}\mathsf{r}_{jki}}{\omega_i - \omega_j - \omega_k} \right. \\ & \left. - \frac{\mathsf{g}_{ii,k}\mathsf{f}_{jjk} + \mathsf{f}_{iik}\mathsf{g}_{jj,k}}{\omega_k}\right] \end{aligned} \tag{S42}$$

# References


(S1) Podolsky, B. Quantum-mechanically correct form of Hamiltonian function for conservative systems. *Phys. Rev.* **1928**, *32*, 812.

(S2) Wilson, E. B.; Decius, J. C.; Cross, P. C. *Molecular Vibrations: The Theory of Infrared and Raman Vibrational Spectra*; McGraw-Hill Book Company, 1955.

(S3) Peng, C.; Ayala, P. Y.; Schlegel, H. B.; Frisch, M. J. Using redundant internal coordinates to optimize equilibrium geometries and transition states. *J. Comput. Chem.* **1996**, *17*, 49–56.

(S4) Brandhorst, K.; Grunenberg, J. Efficient computation of compliance matrices in redundant internal coordinates from Cartesian Hessians for nonstationary points. *J. Chem. Phys.* **2010**, *132*, 184101.

(S5) Natanson, G. A. Optimum choice of the reaction coordinate for adiabatic calculations of the tunneling probabilities. *Chem. Phys. Lett.* **1992**, *190*, 209–214.

(S6) Pulay, P.; Fogarasi, G. Geometry optimization in redundant internal coordinates. *J. Chem. Phys.* **1992**, *96*, 2856–2860.

(S7) Grochowski, P. Rotational symmetry of the molecular potential energy in the Cartesian coordinates. *Theor. Chem. Acc.* **2008**, *121*, 257–266.

(S8) Miyazawa, T. Symmetrization of secular determinant for normal vibration calculation. *J. Chem. Phys.* **1958**, *29*, 246–246.
